# Non-proteolytic ubiquitination of Hexokinase 2 by HectH9 controls tumor metabolism and cancer stem cell expansion

**DOI:** 10.1038/s41467-019-10374-y

**Published:** 2019-06-14

**Authors:** Hong-Jen Lee, Chien-Feng Li, Diane Ruan, Jiabei He, Emily D. Montal, Sonja Lorenz, Geoffrey D. Girnun, Chia-Hsin Chan

**Affiliations:** 1 Department of Pharmacological Sciences, Stony Brook University, Stony Brook, NY 11794 USA; 2 Stony Brook Cancer Center, Stony Brook University, Stony Brook, NY 11794 USA; 3 National Institute of Cancer Research, National Health Research Institutes, Tainan, 704 Taiwan; 4 Department of Pathology, Chi-Mei Foundational Medical Center, Tainan, 710 Taiwan; 5 Department of Pathology, Stony Brook University, Stony Brook, NY 11794 USA; 6 Rudolf Virchow Center for Experimental Biomedicine, University of Würzburg, Josef- Schneider-Strasse 2, D-97080 Würzburg, Germany

**Keywords:** Cancer, Molecular biology, Cancer metabolism

## Abstract

Enormous efforts have been made to target metabolic dependencies of cancer cells for developing new therapies. However, the therapeutic efficacy of glycolysis inhibitors is limited due to their inability to elicit cell death. Hexokinase 2 (HK2), via its mitochondrial localization, functions as a central nexus integrating glycolysis activation and apoptosis resilience. Here we identify that K63-linked ubiquitination by HectH9 regulates the mitochondrial localization and function of HK2. Through stable isotope tracer approach and functional metabolic analyses, we show that HectH9 deficiency impedes tumor glucose metabolism and growth by HK2 inhibition. The HectH9/HK2 pathway regulates cancer stem cell (CSC) expansion and CSC-associated chemoresistance. Histological analyses show that HectH9 expression is upregulated and correlated with disease progression in prostate cancer. This work uncovers that HectH9 is a novel regulator of HK2 and cancer metabolism. Targeting HectH9 represents an effective strategy to achieve long-term tumor remission by concomitantly disrupting glycolysis and inducing apoptosis.

## Abstract (web summary)

Cancer cells develop specific metabolic adaptations. Here, the authors show that in prostate cancer models, the ubiquitin ligase Hect9 promotes tumor growth by accelerating glucose metabolism via ubiquitination of Hexokinase 2, a central regulator of glycolysis.

## Introduction

Most tumors increase their glucose metabolism to meet increased energy, biosynthesis and redox needs. Not only does accelerated glucose metabolism represent a hallmark of cancer cells, it also contributes directly to biological processes regulating the growth, dissemination and treatment resistance of tumors^1–3^. The distinct metabolic feature of cancer cells makes targeting metabolism a compelling basis for new cancer therapies. Aerobic glycolysis, also known as the Warburg effect, drives tumorigenesis by rapidly generating ATP to sustain cell hyperproliferation and secreting lactic acid to mitigate immune response in the tumor microenvironment^4,5^. Moreover, it empowers the pentose phosphate pathway that mediates the synthesis of nucleotides, lipids and amino acids for tumor growth. Recent studies have established the cancer-promoting function of mitochondrial oxidative phosphorylation (OXPHOS) by regulating cell growth and redox homeostasis^6^. Moreover, elevated glycolysis and OXPHOS enable epithelial-mesenchymal-transition and cancer stem cell (CSC) phenotypes^7–9^. In view that CSCs are a main cause of tumor development and treatment failure, a better understanding of cancer-specific metabolic liabilities and their regulation in CSCs are key to developing metabolism-targeted cancer therapies.

Hexokinase (HK) catalyzes the first committed step of glucose metabolism by converting glucose to glucose-6-phosphate (G-6-P). This G-6-P then initiates major pathways of glucose utilization including glycolysis, the pentose phosphate pathway and OXPHOS, therefore HK is regarded as a crucial regulator of glucose metabolism. Among HK isoforms, HK1 and HK2 are associated with mitochondria and implicated in cell survival^10^. HK1 is ubiquitously expressed in most mammalian tissues. On the other hand, HK2 expression is limited in most normal tissues but frequently upregulated in various human cancers^11–15^. Animal studies showed that multiple oncogenic drivers, e.g., ErbB2 overexpression, KRAS mutation and loss of Pten, and p53 induce expression of HK2 but not HK1. Systemic ablation of HK2 in genetic mouse models inhibits tumor development without adverse physiological outcomes^16,17^. HK1 and HK2 both bind to the outer mitochondrial membrane via interaction with voltage-dependent anion channel (VDAC). The mitochondrially-bound HKs are in close proximity to the intramitochondrial ATP and consequently promote glycolysis. In addition, the mitochondrial association between HK and VDAC inhibits cellular apoptosis by closing the mitochondrial permeability transition pores and preventing cytochrome c release^18,19^. While HK2 shuttles from the cytoplasm to the mitochondria in reaction to environmental and metabolic stress, HK1 localization is less sensitive to these factors and functions as a housekeeping protein of glucose metabolism^11,20^. Increased mitochondrial-bound HK2 exerts dual cancer-promoting effects by concomitantly promoting glycolysis and inhibiting apoptosis. Despite of the therapeutic potential, strategies that directly and selectively target HK2 are not yet conceivable. It is of immense interest to decipher the regulatory machinery and mechanism selective for HK2’s mitochondrial transportation.

Unlike lysine (K) 48-linked ubiquitination that leads to protein degradation, K63-linked ubiquitination activates kinase and transcription factors, regulates protein trafficking and targets transmembrane proteins to the multivesicular bodies and lysosomal degradation^21,22^. The HectH9 (also named ARF-BP1, HUWE1, and MULE) E3 ligase is shown to orchestrate diverse biological functions by ubiquitination. HectH9 downregulates the p53 tumor suppressor by inducing its K48-linked ubiquitination^23–25^. HectH9 promotes tumorigenesis by activating K63-linked ubiquitination and transcriptional activity of Myc^26^. Moreover, HectH9 is upregulated upon hypoxia and is co-upregulated with Hif1α in tumor tissues^27^. Despite hypoxia is a pervasive environmental activator of tumor glycolysis, HectH9’s role in metabolic responses remains elusive. In this study, we use a combination of stable isotope tracer approach and functional metabolic analysis to discover that HectH9 is a novel activator of glucose metabolism. We show that HectH9 promotes HK2’s K63-linked ubiquitination: this post-translational modification regulates HK2’s localization to mitochondria and the subsequent functions in glycolysis induction and apoptosis prevention. Our results also illustrate that blocking the HectH9/HK2 pathway inhibits ROS-mediated CSC expansion and tumor development, providing a new roadmap to combat drug-resistant tumors.

## Results

### HectH9 regulates glucose metabolism in prostate cancer

**Fig. 1:**
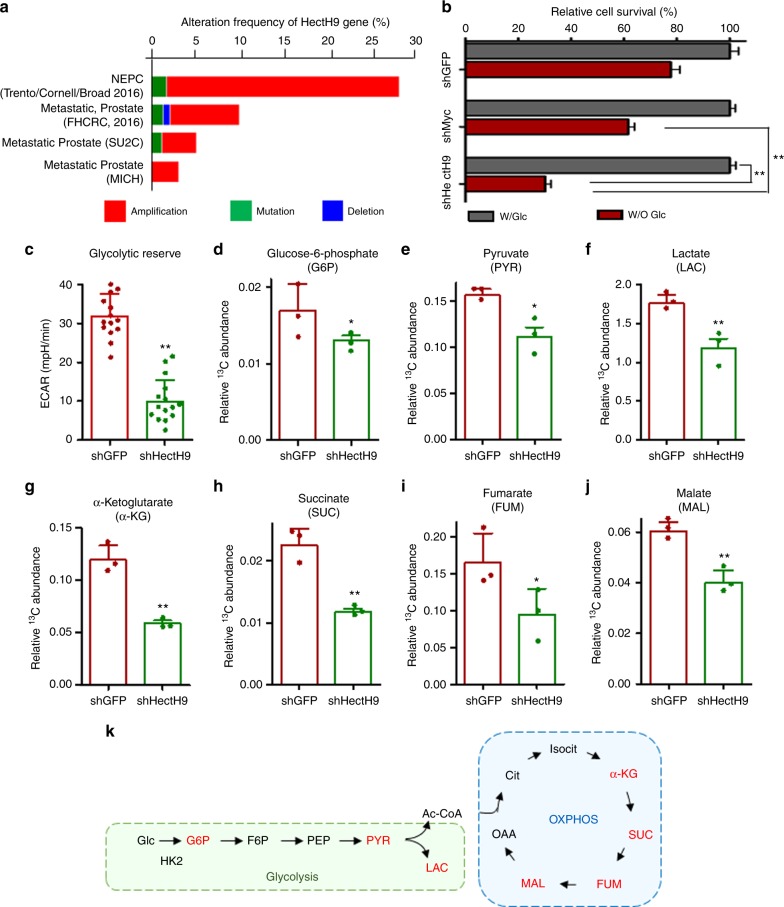
Tumor-associated HectH9 is a novel regulator of glucose metabolism. **a** cBioportal was used to access TCGA data for HectH9 (*Huwe1*) gene alteration from four different studies of human prostate cancer. Frequencies of HectH9 gene amplification are shown in red. (**b**) Cell viability assay in PC-3 cells stably infected with lentiviruses expressing shRNA targeting GFP, Myc or HectH9 in glucose-containing and -deprived medium (*n* = 3). **c** The extracellular acidification rate (ECAR), was measured by using a Seahorse Bioanalyzer in PC-3 cells infected with lentiviruses expressing shRNA targeting GFP or HectH9 (*n* = 15). **d–j** Relative abundance of ^13^C-labeled glucose-6-phosphate (**d**), pyruvate (**e**), lactate (**f**), α-ketoglutarate (**g**), succinate (**h**), fumarate (**i**) and malate (**j**) were determined by GC-MS in PC-3 cells infected with lentiviruses expressing shRNA targeting GFP or HectH9 (*n* = 3). Results in **b–j** are presented as mean value ± SD; *p < 0.05, **p < 0.01, by Student’s *t*-test. Experiments were performed at least twice. **k** Schematic illustration depicts the metabolic changes (labeled in red) in glycolysis pathway and oxidative phosphorylation (OXPHOS) regulated by HectH9

Analysis of The Cancer Genome Atlas (TCGA) dataset by cBioPortal demonstrated that the HectH9 gene is upregulated in advanced prostate cancer (Fig. 1a). A recent report demonstrated that HectH9 is highly regulated by hypoxia^27^, a pervasive environmental stimulus of glucose metabolic reprogramming. Indeed, we showed that HectH9 expression in prostate cancer cells is upregulated upon hypoxia (Supplementary Fig. 1a). To study HectH9’s potential role in glucose metabolism, we examined if HectH9 is involved in the glucose dependency of prostate cancer cells. Our data showed that HectH9 deficiency greatly impaired cell survival under glucose deprivation (Fig. 1b and Supplementary Fig. 1b). Notably, the cell growth inhibitory effect caused by genetic silencing of HectH9 is greater than that of Myc, a known HectH9 substrate and a metabolic regulator (Fig. 1b and Supplementary Fig. 1b, c). To determine how HectH9 participates in cancer cell metabolism, we analyzed its impact on the extracellular acidification rate (ECAR), an indicator of glycolytic efficiency^28^. As shown in Fig. 1c, depletion of HectH9 greatly lowered the ECAR. Consistently, HectH9 depletion inhibited the production of lactate, the end product and readout of the glycolytic pathway (Supplementary Fig. 1d). The level of glycolysis inhibition by HectH9 depletion was greater than that of Myc (Supplementary Fig. 1d). Moreover, double deficiency in HectH9 and Myc led to greater glycolysis inhibition than single deficiency in HectH9 and Myc (Supplementary Fig. 1e). These results imply that HectH9 regulates glucose utilization through other downstream target beside Myc. We next conducted metabolite profiling by using a [U6-^13^C_6_]-glucose tracer approach in combination with gas chromatography–mass spectrometry (GC-MS) analysis to pinpoint at which step HectH9 alters glucose metabolism. Our data revealed that HectH9 deficiency globally downregulated the abundance of multiple glycolytic intermediates, including G-6-P, pyruvate and lactate (Fig. 1d–f and Supplementary Fig. 1f). In addition, multiple metabolites involved in OXPHOS, including α-ketoglutarate, succinate, fumarate and malate were downregulated upon HectH9 knockdown (Fig. 1g–j). Taken together, these data uncover HectH9’s crucial role in glucose metabolism (Fig. 1k).

### HectH9 mediates K63-linked ubiquitination of HK2

HK2 is a cancer-associated isoenzyme catalyzing the first rate-limiting step of glucose metabolism. It serves as a critical nexus of integration among energy production, preservation of mitochondrial integrity and cell survival^10^. In light of HK2’s restricted expression in cancer cells and essential role in tumor development^15–17,29,30^, targeting HK2 is considered an attractive therapeutic strategy.

**Fig. 2:**
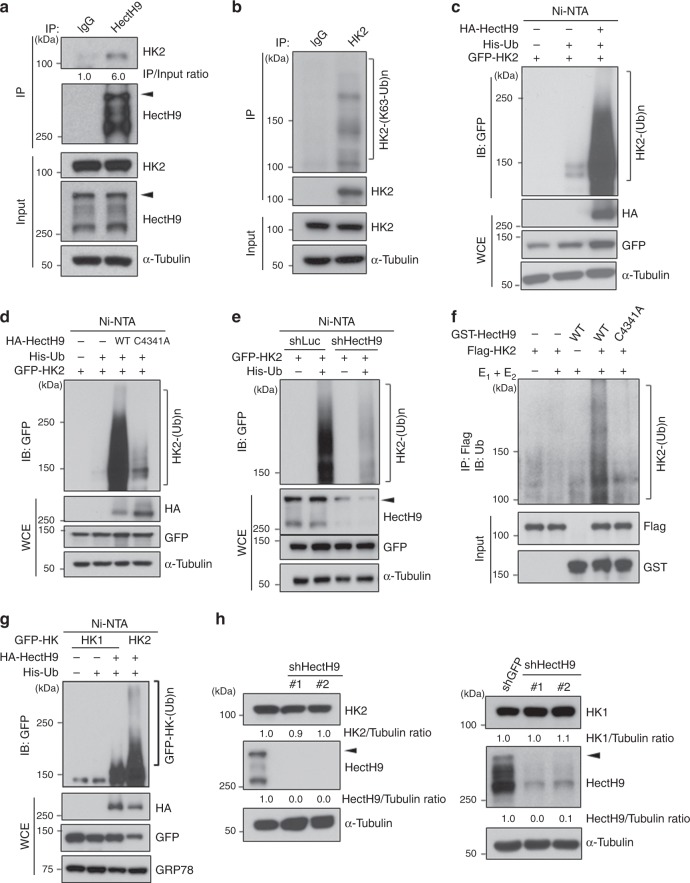
HectH9 promotes K63-linked ubiquitination of HK2. **a** PC-3 cell lysates were harvested for immunoprecipitation (IP) with IgG or anti-HectH9 antibody, followed by immunoblot (IB) analysis with indicated antibodies. The ratio of immunoprecipitated HK2 to total HK2 levels in the input was quantified using ImageJ software. Arrowhead indicates the full-length of HectH9 and signals underneath represent HectH9 fragments. **b** PC-3 cell lysates were harvested for IP with IgG or anti-HK2 antibody, followed by IB analysis with indicated antibodies. **c** and **d** In vivo ubiquitination assay of HK2 by HectH9. HEK293T cells transfected with indicated plasmids were harvested for ubiquitination assay. Cells were lysed by denatured buffer. The ubiquitinated proteins were precipitated with nickel (Ni)-NTA beads and subjected to IB analysis. NTA indicates nickel bead precipitate; WCE indicates whole-cell extracts. **e** HEK293 cells with Luciferase or HectH9 knockdown were transfected with indicated constructs and harvested for in vivo ubiquitination assay. **f** Purified catalytically active form (WT) or defective mutant (C4341A) of GST-HectH9 proteins were incubated with adenosine triphosphate, E1 (Ube1) and E2 (Ubc13), with or without purified Flag-HK2 for in vitro HK2 ubiquitination assay. **g** HEK293T cells transfected with indicated constructs were harvested for in vivo ubiquitination assay. **h** IB analysis of HK2 and HK1 expression in PC-3 cells with GFP or HectH9 knockdown. Two HectH9 lentiviral shRNAs were used in this assay. The relative intensity of protein expression as indicated was quantified with ImageJ software and normalized to α-Tubulin expression. Immunoblots were performed three times

Our finding that HectH9 regulates the glucose conversion to G-6-P suggests potential crosstalk between HectH9 and HK2. Indeed, co-immunoprecipitation assays revealed endogenous interaction between HectH9 and HK2 in prostate cancer cells (Fig. 2a and Supplementary Fig. 2a). The HectH9 E3 ligase has been shown to regulate protein substrates by triggering either K63-linked or K48-linked ubiquitination^31^. We therefore sought to determine which form of ubiquitin linkage is responsible for HectH9-mediated HK2 ubiquitination. As shown in Fig. 2b and Supplementary Fig. 2b, endogenous HK2 underwent K63-linked, but not K48-linked, ubiquitination. Overexpression of the catalytically active HectH9 robustly promoted HK2 ubiquitination (Fig. 2c), whereas the E3 ligase activity dead mutant (C4341A) failed to do so (Fig. 2d and Supplementary Fig. 2c), indicating that HectH9-mediated HK2 ubiquitination depends on its E3 ligase activity. Conversely, HK2 ubiquitination was abolished upon HectH9 deficiency (Fig. 2e). The catalytically active form but not the C4341A mutant of HectH9 in collaboration with Ubc13, an E2 enzyme for conjugating K63-linked ubiquitination, robustly triggered HK2 ubiquitination in vitro (Fig. 2f), underscoring that HectH9 is a bona fide E3 ligase of HK2. In addition, we examined whether HectH9 promotes ubiquitination of HK1, the house keeping isoform. Our data indicated that HectH9 preferentially triggered ubiquitination of HK2 over HK1 (Fig. 2g). These findings together suggest that HectH9-mediated K63-linked ubiquitination is selective for HK2 regulation and that HectH9 works through HK2 in regulating glycolysis.

### HectH9 regulates the localization and function of HK2

**Fig. 3:**
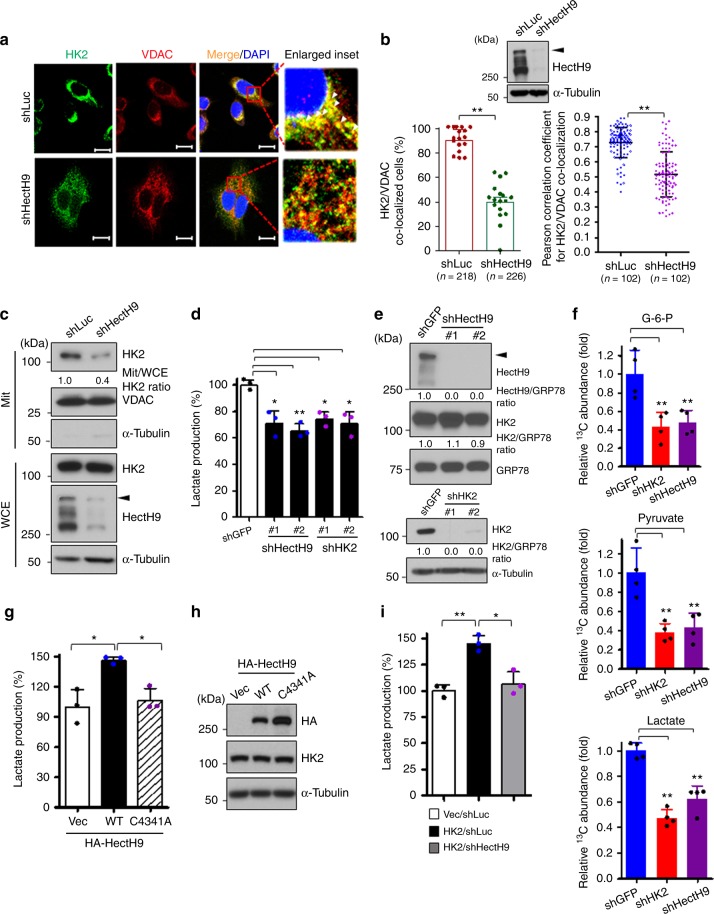
HectH9 regulates HK2 localization to mitochondria and HK2-mediated glycolysis. **a** Immunofluorescence image for HK2/VDAC colocalization in Luciferase-knockdown and HectH9-knockdown PC-3 cells (from three biological replicates). VDAC and DAPI was used as markers for mitochondria and nucleus, respectively. White arrowheads show co-localization of HK2/VDAC at mitochondria. Scale bar represents 10 μm. **b** IB analysis of HectH9 expression in PC-3 cells with Luciferase or HectH9 knockdown (upper). Quantification analyses of HK2/VDAC co-localization in PC-3 cells with Luciferase or HectH9 knockdown (lower). The lower left panel shows the percentage of counted cells with HK2/VDAC colocalization. 17 random fields of view in shLuc and shHectH9 groups that account for 218 and 226 cells, respectively, were counted. The lower right panel shows that the HK2/VDAC correlation per cell was determined using the Pearson’s correlation coefficient. 102 cells were counted in each group from three biological replicates. **c** Biochemical fractionation for HK2 subcellular localization in Luciferase-knockdown and HectH9-knockdown PC-3 cells. VDAC and α-Tubulin were used as markers for mitochondrial and cytosolic fractions, respectively. The ratio of mitochondrial HK2 to total HK2 levels in WCE was quantified by ImageJ. **d** Lactate production in PC-3 cells with GFP, HectH9 or HK2 knockdown. (*n* = 3). **e** IB analysis for HectH9 and HK2 expression in PC-3 cells with GFP, HectH9, or HK2 knockdown. The relative intensity of protein expression as indicated was quantified with ImageJ and normalized to GRP78 or α-Tubulin expression. **f** Relative abundance of ^13^C-labeled glucose-6-phosphate, pyruvate and lactate were determined by GC-MS in PC-3 cells with GFP, HK2 or HectH9 knockdown (*n* = 4). The relatively abundance was normalized to that in GFP-knockdown cells. **g** and **h** Lactate production (**g**) and IB analysis (**h**) were carried out in HeLa cells transfected with indicated plasmids. **i** Lactate production was measured in HK2-depleted HeLa cells, followed by restoration of vector control (Vec), WT HK2 or WT HK2 plus HectH9-knockdown. (*n* = 3). Results in **b**, **d**, **f**, **g** and **i** are presented as mean value ±SD; *p<0.05, **p<0.01, by Student’s t-test. Experiments were performed at least twice in triplicates. Immunoblots were performed three times

We sought to delineate the underlying mechanism by which HectH9 regulates HK2. An immunoblot assay showed that HectH9 deficiency did not affect the level of either HK2 or HK1 isoform, excluding HectH9’s role in protein stabilization of HKs (Fig. 2h). HK2 is known to localize to mitochondria via association with VDAC at the mitochondrial outer membrane. Mitochondrial-bound HK2 works in concert with ATP cofactors to catalyze glucose phosphorylation to initiate glycolysis^32^. We next examined if HectH9 regulates HK2 by changing its subcellular localization. Immunofluorescent staining illustrated that the majority of HK2 emerged in a punctuate distribution and was co-localized with VDAC in prostate cancer cells (Fig. 3a, b). Depletion of HectH9 impaired HK2’s mitochondrial localization and led to its cytosolic diffusion (Fig. 3a, b). To further substantiate this finding, we performed a biochemical fractionation assay and showed that HectH9 deficiency attenuated HK2 localization to mitochondria (Fig. 3c). Along with these observation, we showed that the mitochondria-bound HK2 preferentially underwent K63-linked ubiquitination than the cytosolic HK2 (Supplementary Fig. 2d). Moreover, HectH9 depletion inhibited the lactate production and ECAR to a similar degree as HK2 knockdown in prostate cancer cells (Fig. 3d, e and Supplementary Fig. 2e). Glucose tracing experiments showed that HectH9 depletion downregulated the abundance of multiple glycolytic intermediates, including glucose-6-posphate, pyruvate and lactate, to a similar degree as HK2 knockdown (Fig. 3f). In addition, HectH9 knockdown phenocopies HK2’s effects on downregulating glucose-derived OXPHOS metabolites such as α-KG, succinate, fumarate and malate (Supplementary Fig. 2f). A lactate production assay showed that the HectH9/HK2 axis regulates the aerobic glycolysis of HeLa cells (Supplementary Fig. 2g, h). Ectopic expression of HectH9 promoted lactate production, whereas the catalytic dead C4341A mutant exhibited impairment to do so (Fig. 3g, h). These observations, along with the GC-MS analysis data, uncovered HectH9’s novel function in glycolysis activation. The induced lactate production resulting from HK2 overexpression was attenuated upon HectH9 deficiency (Fig. 3i), establishing a functional link between HectH9 and HK2 in promoting cancer glycolysis.

### HectH9 depletion induces apoptosis upon glucose deprivation

**Fig. 4:**
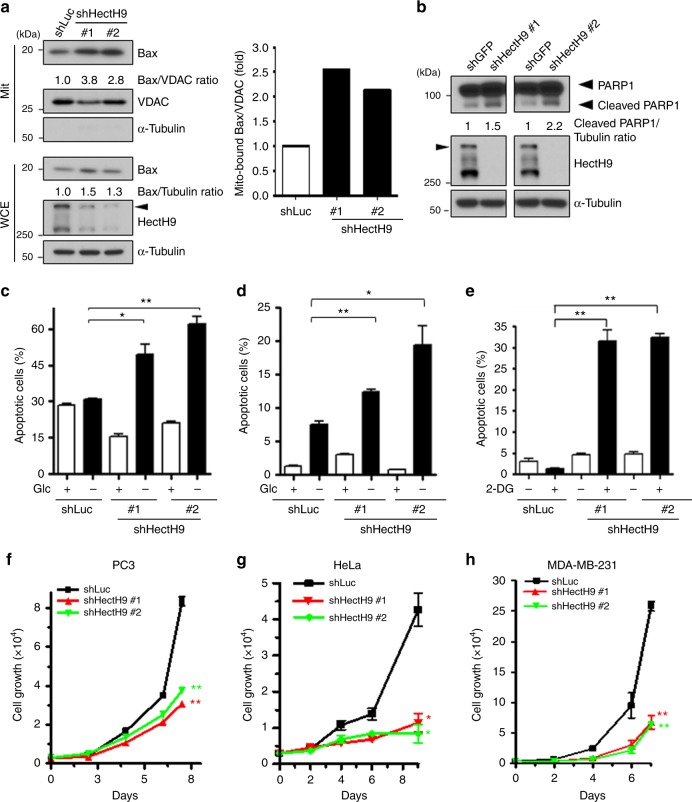
HectH9 deficiency triggers cellular apoptosis under glucose deprivation. **a** Biochemical fractionation assay for Bax mitochondrial localization in PC-3 cells with Luciferase or HectH9 knockdown is shown in the left panel. VDAC and α-Tubulin were used as markers for mitochondrial and cytosolic fractions, respectively. The relative intensity of mitochondrial-bound Bax quantified with ImageJ software and normalized to mitochondrial VDAC levels in the corresponding cells is shown in the right. The experiment was performed twice. **b** IB analysis of cleaved PARP1 expression in PC-3 cells with GFP or HectH9 knockdown. The relative intensity of cleaved PARP1 expression was quantified with ImageJ software and normalized to α-Tubulin expression. The experiment was performed twice. **c** and **d** The percentage of apoptotic cells was determined by annexin V and propidium iodide (PI) staining in Luciferase- knockdown and HectH9-knockdown PC-3 cells (**c**) or MDA-MB-231 cells (**d**) cultured in media with or without glucose (Glc) (*n* = 3). **e** The percentage of apoptotic cells was determined by annexin V and PI staining in Luciferase-knockdown and HectH9-knockdown PC-3 cells in the presence of vehicle or 2-DG (*n* = 3). **f–h** Cell growth assays in PC-3 (**f**), HeLa (**g**) and MDA-MB-231 (**h**) cells with Luciferase or HectH9 knockdown (*n* = 3). Results in **c–h** are presented as mean value ± SD; *p < 0.05, **p < 0.01, by Student’s *t*-test. These experiments were performed three times in triplicates. Immunoblots were performed three times

Mitochondria play a critical role in cell survival in part through restraining pro-apoptotic protein Bax cytosolic localization. In response to death stimuli (e.g., withdrawal of growth factors or nutrition), Bax translocates to mitochondria, binding with Bak and consequently leads to PARP1 cleavage and cellular apoptosis^33,34^. Mitochondria-bound HK2, through interaction with VDAC, releases Bax from the mitochondria to the cytosol and thus inhibits apoptosis^35,36^. Our data about HectH9-regulated HK2 trafficking prompted us to study HectH9’s role in mitochondria-dependent apoptosis. We found that ablating HectH9 facilitated the localization of Bax to the mitochondria and PARP1 cleavage (Fig. 4a, b). Furthermore, HectH9 deficiency augmented cellular apoptosis upon glucose deprivation in multiple cancer cell lines (Fig. 4c, d and Supplementary Fig. 3a, b).

The synthetic glucose analog and competitive glycolysis inhibitor 2-deoxy-D-glucose (2-DG) shuts down G-6-P production for subsequent glycolytic cascades. Despite its promising effects on growth suppression, 2-DG has limited clinical success as a single agent due to its inability to trigger cell death programs. Annexin V staining showed that while 2-DG alone barely induced apoptosis as expected, its combination with HectH9 deficiency greatly prompted cancer cells to undergo apoptosis even in p53-null prostate cancer cells (Fig. 4e and Supplementary Fig. 3c, d). Our results suggest that HectH9 inhibition could potentially be used to improve the anti-cancer efficacy of glycolytic inhibitors. In addition to prostate cancer, previous reports and the Human Protein Atlas study (HPA002548) have demonstrated that HectH9 protein is overexpressed in breast and cervical tumors^26,37^. Cell viability assays showed that HectH9 knockdown markedly impaired cell survival in prostate, breast and cervical cancer cells regardless of p53 status (Fig. 4f–h and Supplementary Fig. 3d-f). Of note, HectH9 ablation does not cause p53 stabilization in p53-proficeint cancer cells (Supplementary Fig. 3e, f). These results imply that deficiency in HectH9 triggers p53-independent apoptosis by preventing mitochondrial transport of HK2.

### K63-linked ubiquitination regulates the functions of HK2

**Fig. 5:**
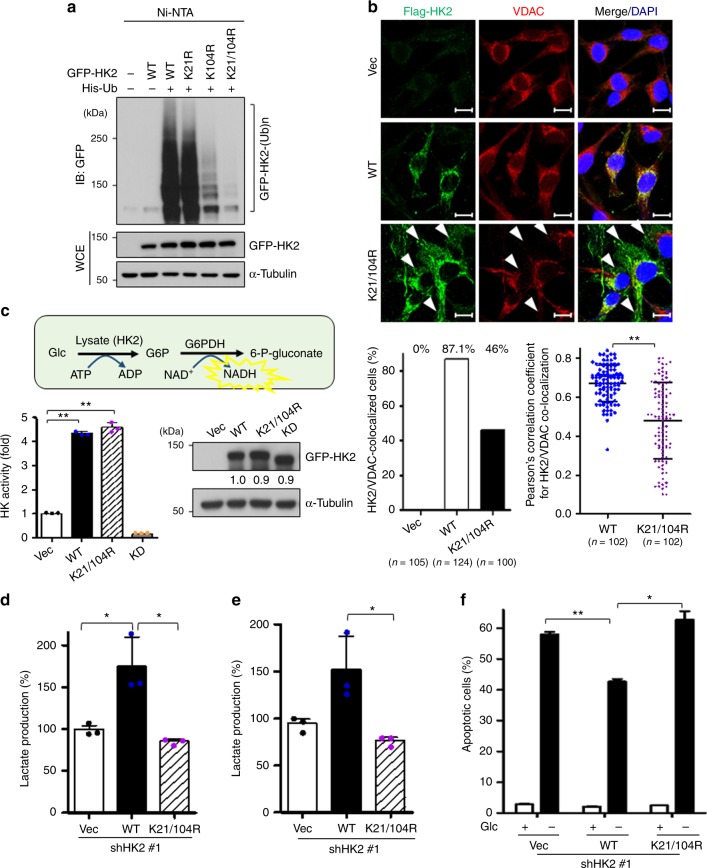
K63-linked ubiquitination regulates the mitochondrial localization and functions of HK2. **a** In vivo ubiquitination assay in HEK293T cells transfected with or without His-Ub, along with WT, K21R, K104R, and K21/104 R mutants of GFP-HK2. **b** MDA-MB-231 cells infected with viruses expressing vector control (Vec), WT or the K21/104 R mutant of Flag-HK2 were fixed and subjected to immunofluorescence staining with indicated antibodies and DAPI. Representative images of HK2 and VDAC subcellular localization are shown in the upper panel. White arrowheads indicate that the K21/104 R mutant of HK2 diffuses in cytosol and is not co-localized with VDAC on mitochondrial outer membrane. Scale bar represents 10 μm. Quantification results for HK2/VDAC co-localization are shown in the lower panels. The lower left panel shows the percentage of counted cells with co-localized HK2 and VDAC. At least 100 cells were counted in each group from three biological replicates. The lower right panel shows that the HK2/VDAC correlation per cell was determined using the Pearson’s correlation coefficient. 102 cells were counted in each group from three biological replicates. **c** HK2 kinase activity was measured in HEK293T cells transfected with indicated plasmids. KD represents kinase dead (*n* = 3). Schematic illustration and quantification of the HK2 activity assay are shown in the upper and lower panels, respectively. IB analysis for protein expression of vector control, WT and various mutants of GFP-HK2 is shown in the right panel. The relative intensity of GFP-HK2 protein expression was quantified with ImageJ software and normalized to α-Tubulin expression. **d** and **e** Lactate production was measured in MDA-MB-231 (**d**) and HeLa cells (**e**) with HK2 knockdown, followed by ectopic expression of vector control, WT or the K21/104 R mutant of HK2 (*n* = 3). **f** The apoptotic cell percentage was determined by annexin V and PI staining in the absence or presence of glucose (Glc) in MDA-MB-231 cells with HK2 knockdown, followed by ectopic expression of vector control, WT or the K21/104R mutant of HK2 (*n* = 3). Results in **b, d–f** are presented as mean value ± SD; *p < 0.05, **p < 0.01, by Student’s *t*-test. Experiments were performed at least twice in triplicates. Immunoblots were performed three times

To characterize the function of K63-linked ubiquitination of HK2, we sought to identify the K residue(s) on HK2 where K63-linked ubiquitination takes place. Earlier studies showed that the first 21 amino acids present in the N-terminus of HK2 regulate the mitochondrial transportation of HK2^38^. Since K21 is the only evolutionarily conserved K residue present in the N-terminal region (Supplementary Fig. 4a), we performed site-directed mutagenesis of K21 to test its role in HK2 protein trafficking. Mutation on K21 to arginine slightly impaired the ubiquitination and mitochondrial localization of HK2 (Fig.5a and Supplementary Fig. 4b, c). This result led us to look for other K residues critical for HK2 ubiquitination and mitochondrial trafficking. Sequence analysis revealed high similarity between the sequence encompassing the K104 residue (QK_104_VE) and that harboring the K21 residue (QK_21_VD) (Supplementary Fig. 4a), suggesting that K104 could be another potential regulatory site responsible for HK2’s ubiquitination and transportation. A single mutation on K104 to arginine significantly mitigated ubiquitination and mitochondrial localization of HK2 (Fig. 5a and Supplementary Fig. 4b, c). Double mutation on K21 and K104 markedly attenuated HK2 ubiquitination (Fig. 5a). The ubiquitination-defective mutant (K21/K104R) of HK2 had impaired mitochondrial transport and a diffused appearance in the cytosol in various cancer cell lines (Fig. 5b and Supplementary Fig. 4b, c). Moreover, an IP assay demonstrated that overexpression of WT HK2 increased the association to VDAC, whereas the association between VDAC and HK2 was impaired upon defective HK2 ubiquitination (Supplementary Fig. 4d), underscoring that K63-linked ubiquitination of HK2 promotes HK2 localization to the mitochondria by increasing HK2 interaction with VDAC. It is worth noting that the ubiquitation event does not significantly affect the protein stability of HK2 (Supplementary Fig. 4e). HK2 phosphorylation by Akt has been shown to participate in HK2 binding to mitochondria^39,40^. We found that double, but not single mutation on K21 and K104 attenuated HK2 phosphorylation by Akt (Supplementary Fig. 4f), suggesting that K63-linked ubiquitination of HK2 exerts its effects on HK2 binding to mitochondria in part through Akt-driven HK2 phosphorylation. Of note, the K104 site used for conjugating K63-linked ubiquitination of HK2 is absent in HK1, supporting our earlier finding that HectH9-mediated ubiquitination is selective for HK2 (Fig. 2g and Supplementary Fig. 4g).

We also examined whether K63-linked ubiquitination also affects HK2’s enzymatic activity. The kinase activity assay showed that HK2 depletion effectively attenuated the enzymatic activity (Supplementary Fig. 4h), whereas the ubiquitination-defective mutant of HK2 retained its kinase activity at a similar level as WT HK2 (Fig. 5c). These data underscore that the defect in HK2 ubiquitination does not destroy HK2’s protein activity or its structure but selectively regulates protein trafficking of HK2. We next compared the biological effect of WT and the ubiquitination-defective mutant of HK2. WT and the K21/K104R mutant of HK2 were restored in the cancer cells infected with viruses containing a shRNA targeting the 3’-UTR of HK2. Our results showed that restoring WT HK2 promoted HK2’s functions in glycolysis and anti-apoptosis as expected, whereas the K21/K104R HK2 mutant lost the abilities (Fig. 5d–f and Supplementary Fig. 4i, j). These findings underscore the importance of K63-linked ubiquitination in regulating HK2’s mitochondrial trafficking and functions.

### The HectH9/HK2 pathway regulates CSCs via ROS production

**Fig. 6:**
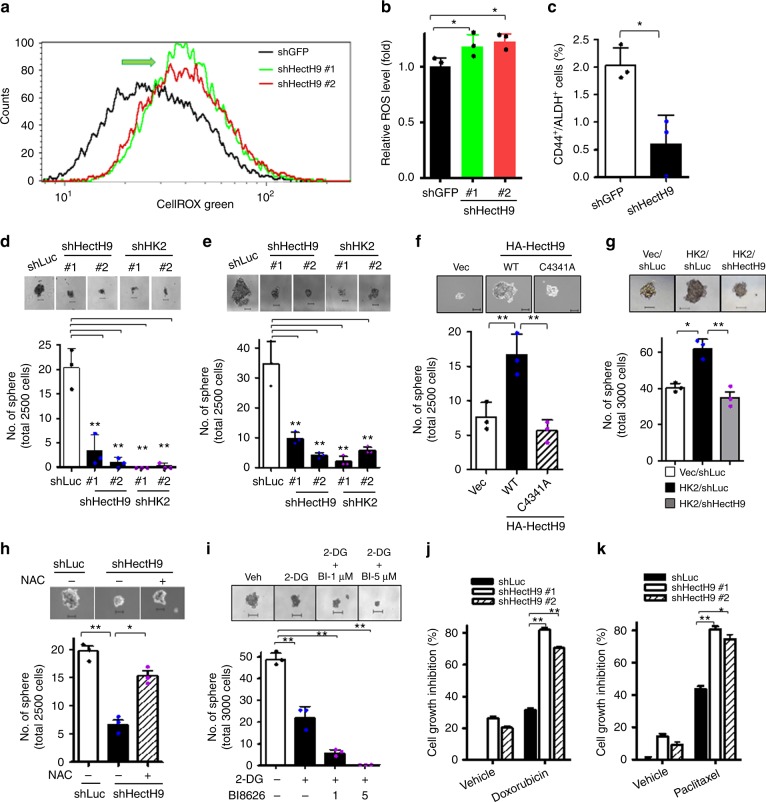
Deficiency in HectH9 or HK2 inhibits CSC self-renewal via ROS production. **a** Representative image of flow cytometry analysis for ROS production in PC-3 cells with GFP or HectH9 knockdown. Green arrow indicates higher ROX level in HectH9-knockdown PC-3 cells. **b** Quantitative results for ROS production in PC-3 cells with GFP or HectH9 knockdown. Data were collected from three independent experiments. **c** Populations of CD44^+^/ALDH^+^ cells were determined by flow cytometry analysis in PC-3 cells with GFP or HectH9 knockdown (*n* = 3). **d** and **e** Tumor sphere formation assays in PC-3 (**d**) and HeLa (**e**) cells with Luciferase, HectH9 or HK2 knockdown (*n* = 3). Scale bar represents 100 μm. **f** Tumor sphere formation assay in HeLa cells infected with lentiviruses containing shRNA targeting the 3’-UTR of HectH9, followed by restoration with vector control, catalytically active form (WT) or defective mutant (C4341A) of HA-HectH9 (*n* = 3). Scale bar represents 100 μm. **g** Tumor sphere formation assay in HeLa cells infected with viruses expressing vector control or HK2 overexpression, followed by Luciferase or HectH9 knockdown (*n* = 3). Scale bar represents 100 μm. **h** Tumor sphere formation assay in PC-3 cells with Luciferase or HectH9 knockdown in the absence and presence of NAC (*n* = 3). Scale bar represents 100 μm. **i** Tumor sphere formation assay in PC-3 cells incubated with vehicle, 2-DG alone or 2-DG and BI8626 in combination for 12 days (*n* = 3). **j** and **k** Cell growth inhibition assay in PC-3 cells with Luciferase or HectH9 knockdown in the absence and presence of doxorubicin for 96 h (**j**) or paclitaxel for 48 h (**k**) (*n* = 3). Cell numbers were counted by using a hemocytometer. Results are presented as mean value ± SD; *p < 0.05, **p < 0.01, by Student’s *t*-test. Experiments were performed at least twice in triplicates

CSCs possess unlimited self-renewal ability and multipotency. CSC activation and expansion are vital for cancer initiation, progression and therapy resistance^41^. Accumulating evidence suggests that ROS generation inhibits CSC self-renewal^42–44^. Mitochondrial HK2 is known to reduce ROS production by closing mitochondrial permeability transition pores^45,46^. Having shown that HectH9’s crucial role in transporting HK2 to mitochondria, we examined HectH9’s role in CSC self-renewal and ROS generation. We found HectH9 depletion increased ROS production (Fig. 6a, b). Moreover, HectH9 deficiency profoundly reduced the pool size of CSCs in multiple cancer cell types (Fig. 6c and Supplementary Fig. 5a, b). In line with these observations, HectH9 knockdown reduced the number and size of tumor spheres to a level similar to HK2 deficiency (Fig. 6d, e and Supplementary Fig. 5c, d), signifying an indispensable role of HectH9 and HK2 in CSC self-renewal. Conversely, defective CSC expansion mediated by HectH9 depletion was rescued by restoration of the catalytically active form but not the E3 ligase dead mutant of HectH9 (Fig. 6f and Supplementary Fig. 5e). In addition, increased HK2 expression promoted CSC self-renewal (Fig. 6g and Supplementary Fig. 5f), elucidating that HK2 is an upstream activator of CSC expansion. To ascertain whether HectH9 regulates tumor sphere formation through HK2, we silenced endogenous HectH9 expression in HK2-overexpressing cells and found that HK2-enhanced CSC self-renewal ability was abolished upon HectH9 depletion (Fig. 6g and Supplementary Fig. 5f). ROS scavenging by N-acetylcysteine reverted the phenotypes, including diminished number and size of tumor spheres caused by HectH9 or HK2 depletion (Fig. 6h and Supplementary Fig. 5g, h). These findings suggest that ROS mediates HectH9 and HK2’s function in CSC expansion. Furthermore, these observations uncover an indispensable role of the glycolytic enzyme HK2 and its upstream activator HectH9 in CSC regulation. We then examined the effect of the HectH9 inhibitor BI8626^47^ on tumor sphere growth in the presence of 2-DG. As expected, we found that 2-DG alone effectively inhibited the formation of prostate tumor spheres. Intriguingly, 2-DG in combination with BI8626 further reduced the sphere size and number (Fig. 6i and Supplementary Fig. 5i), supporting the notion that HectH9, by ROS inhibition, can regulate CSCs independently of glycolysis.

The existence of CSCs poses a major hurdle for tumor eradication^41^. CSCs are intrinsically resistant to cytotoxic chemotherapy, thereby causing tumor recurrence and metastasis post treatment. Our result that disrupting the HectH9/HK2 pathway mitigated CSC expansion suggests that HectH9 is a potential determinant of chemosensitivity. Indeed, HectH9 knockdown sensitized cancer cell response to doxorubicin and paclitaxel in various cancer cells (Fig. 6j, k and Supplementary Fig. 5j, k), highlighting that targeting HectH9/HK2 axis could be a new approach for restoring cancer cells’ susceptibility to currently used chemotherapy.

### HectH9 deficiency inhibits tumor metabolism and progression

**Fig. 7:**
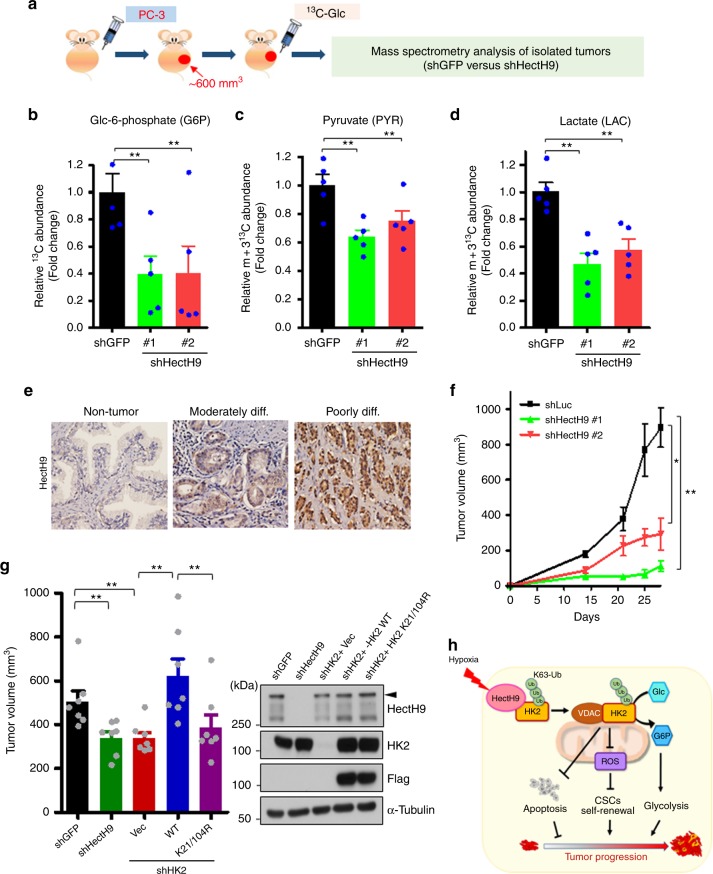
HectH9 deficiency inhibits tumor metabolism and progression. **a** A schematic illustration depicting the process of metabolite profiling in HectH9-proficeint and HectH9-deficient PC-3-derived prostate tumors by GC-MS. **b**–**d** Relative abundances of ^13^C-labeled glucose-6-phosphate (**b**), pyruvate (**c**) and lactate (**d**) were quantified by GC-MS (*n* = 5). **e** Representative images of consecutive tissue sections for histological analyses of HectH9 expressions in normal tissues and prostate tumors with moderately or poorly differentiation status (*n* = 225). **f** In vivo primary tumor growth derived from PC-3 cells with Luciferase or HectH9 knockdown. Cells were injected subcutaneously into the right flanks of nude mice and tumorigenesis was monitored (*n* = 5 in each group). **g** In vivo primary tumor growth derived from MDA-MB-231 cells with HectH9 or HK2 depletion, as well as HK2-depleted MDA-MB-231 cells restored with vector alone (pBabe), WT or K21/104 R of Flag-HK2. Cells were injected into the mammary fat pads of nude mice and tumorigenesis was monitored. Quantitative results of tumor volumes at day 31 are shown in the left (*n* = 7 in each group). IB analysis of HectH9 and HK2 expression in MDA-MB-231 cells with GFP, HectH9 and HK2-depletion, as well as HK2-depleted cells restored with vector alone (pBabe), WT or K21/104 R of Flag-HK2 is shown in the right. **h** The working model depicts that hypoxia-induced HectH9, by promoting K63-linked ubiquitation of HK2, contributes to tumor progression via concomitant glycolysis elevation and apoptosis inhibition in cancer cells, as well as enhanced self-renewal of CSCs mediated by ROS blockade in tumors. Results in **b–d, f** and **g** are presented as mean value ± SEM; *p < 0.05, **p < 0.01, by Student’s *t*-test

To examine HectH9’s role in reprogramming glucose metabolism in vivo, we characterized metabolite changes in prostate tumors upon HectH9 depletion. [U6-^13^C_6_]-glucose tracer was injected into mice bearing prostate xenografts with proficient or deficient HectH9 (Fig. 7a). Tumor xenografts were then isolated for metabolite extraction and profiling. GC-MS analysis revealed that the production of G-6-P was greatly inhibited in HectH9-depleted tumors (Fig. 7b), supporting the notion that HectH9 is an upstream activator of HK2. In addition, HectH9 ablation reduced the abundance of newly produced pyruvate and lactate in vivo (Fig. 7c, d). Despite HectH9’s effect on α-ketoglutarate, it did not have much effect on downstream metabolites involved in OXPHOS, such as malate (Supplementary Fig. 6a, b). These results together illustrate that HectH9 regulates metabolic reprogramming toward the aerobic glycolytic pathway in vivo.

To assess the pathological relevance of the HectH9 in vivo, we analyzed the protein expressions of HectH9 and its relationship to disease progression and malignancy in 225 prostate tumor cases (Table 1). Histopathological analyses showed that HectH9 is overexpressed and that its overexpression is significantly correlated with numerous adverse clinicopathological factors, including increments of tumor stage and poor differentiation of prostate cancer (Fig. 7e, Supplementary Fig. 6c, d and Table 1), supporting the genetic results in TCGA database (Fig. 1a). These in vivo studies indicate that upregulated HectH9 in prostate tumors reprograms cancer metabolism toward glycolysis to promote tumor growth. We next determine whether HectH9 and its mediating HK2 ubiquitination are necessary for tumor development. A tumor xenograft assay showed that targeting HectH9 by genetic silencing profoundly suppressed tumor growth (Fig. 7f). Likewise, inhibiting HK2 ubiquitination mitigated tumor formation (Supplementary Fig. 6e, f). Of note, restoration of WT HK2 in HK2-depleted cells promoted tumorigenesis, whereas the ability of the K21/104 R mutant to promote tumorigenesis was substantially impaired (Fig. 7g). Moreover, we found that the defect in HK2 ubiquitination suppressed tumor growth to a degree similar to HectH9 knockdown (Fig. 7g). These findings collectively provide proof-of-principle evidence that targeting HectH9-mediated HK2 ubiquitination offers a new opportunity for cancer therapy. In addition, we tested the efficacy of the HectH9 inhibitor BI8626 on prostate cancer cell lines (PC3 and LNCaP) and a normal prostate epithelial cell line (PNT1A). The cell viability assay showed that BI8626 effectively inhibited the survival of PC3 and LNCaP cells in a dose-dependent fashion but did not reduce the survival of PNT1A cells (Supplementary Fig. 6g), underscoring that HectH9 inhibition is a potential therapeutic approach.

**Table 1:** Correlations between HectH9 and HK2 expression and clinicopathological parameters in prostate carcinoma

		Case No.	HectH9 Exp. (H-score)	*p*-value	HK2 Exp. (H-score)	*p*-value
Age (years)^a^		225	*r* = −0.075	0.261	*r* = −0.079	0.240
Stage^b^	Low (Stage II)	139	181.9 ± 52.1	<0.001^c^	189.5 ± 46.5	<0.001^c^
	High (Stage III and IV)	86	220.8 ± 50.0		225.1 ± 60.2	
Differentiation^b^	Well/Mod-differentiation (Gleason score ≤ 7)	169	186.2 ± 46.2	<0.001^c^	194.1 ± 47.9	<0.001^c^
	Poorly-differentiation (Gleason score ≥ 8)	56	228.8 ± 64.9		230.2 ± 65.1	
Hexokinase 2 Exp. (H-score)^a^		225	*r* = 0.544	<0.001^c^		

^a^Pearson’s correlation coefficient anaysis; ^b^Mann–Whitney *U* test; ^c^statistically significant

## Discussion

The discovery that tumors acquire dependency on specific metabolic processes has provoked enormous interest in targeting cancer metabolism. Despite so, none of these agents have so far advanced beyond clinical trials^48^. Their primary challenge stems from their inability to induce cell death for long-term tumor remission. For example, 2-DG is among the most advanced cancer metabolism inhibitors in clinical trials (Phase II). Despite an excellent safety profile, 2-DG’s clinical benefit as a single agent is modest, owing to its reversible inhibition of HK2 and inadequacy in eliciting cell death^49–51^. In the current study, we identified that HectH9-promoted HK2 mitochondrial localization is an underlying cause of cancer cells’ resistance to 2-DG and that ablating HectH9 expression synergistically augmented cancer cell sensitivity to 2-DG. Mechanistically, we showed that HectH9 orchestrates HK2 shuttling to mitochondria by non-proteolytic K63-linked ubiquitination. Thus, HectH9-mediated HK2 ubiquitination drives apoptosis resistance, promotes glycolysis and ROS-regulated CSC self-renewal, in turn leading to tumor progression (Fig. 7h). This work reveals HectH9’s previously uncharacterized functions in cancer metabolism and CSC regulation. It also suggests that inhibiting the K63-linked ubiquitination pathway by targeting HectH9 is a new strategy to tackle metabolism-addicted tumors.

HK2 is an attractive drug target against treatment-naïve and -resistant human cancers^16,17,29,52^, sparking various investigations into the underlying molecular basis of HK2 regulation in cancer cells. cMyc and Hif1α transcription factors have been shown to activate gene transcription of HK2^53,54^. HK2 mRNA expression is downregulated by Pten and p53 tumor suppressors. Wang et al. showed that Pten ablation increases HK2 mRNA translation through activation of the Akt-mTOR pathway, while p53 deficiency stabilizes HK2 mRNA through inhibition of miR-143 biogenesis. Double knockout of Pten and p53 upregulates HK2 expression without affecting the HK1 level^17,52^. ErbB2 overexpression and KRAS oncogenic mutations also contribute to the selective HK2 induction in tumor tissues, though the mediating machinery is not completely understood^16,29^. Apart from expression alteration, how HK2 function is activated during tumorigenesis remains obscure. HK2’s dual oncogenic activities in glycolysis and cell survival are mediated by the association between HK2 and VDAC^6,10,55^ and thus disruption of this association may offer new therapeutic opportunities. Earlier studies have shown that Akt activates HK interaction with VDAC and subsequent mitochondrial localization by different mechanisms. For instance, Akt promotes these processes by either directly phosphorylating HKs, or by indirectly suppressing VDAC phosphorylation, a negative regulation for VDAC association with HK2^32,39,56^. Of note, these phosphorylation events did not show the selectivity between HK2 and HK1. In the current study, we discovered that HectH9 preferentially triggered K63-linked ubiquitination of HK2 over HK1. HectH9 deficiency mitigates the HK2-VDAC association at the mitochondria, thereby inducing apoptosis along with glycolysis suppression in cancer cells. These findings together illustrate that K63-linked ubiquitination by HectH9 is a novel mechanism for HK2 activation and cancer progression. The discovered HK2-specific regulation can potentially be exploited for isoform-specific inhibition.

Human and rodent HK2 are both primarily localized at mitochondria. Miyamoto et al. and Roberts et al. previously showed that HK2 phosphorylation at the Thr473 by Akt regulates the mitochondrial association of human and mouse HK2^39,40^. Aside from Akt-mediated HK2 phosphorylation, the current study showed that HectH9-mediated ubiquitination is important for mitochondrial localization of human HK2. HectH9 ubiquitinates human HK2 at K21 and K104 sites. While the primary ubiquitination site K104 in human HK2 is not present in mouse HK2, the minor ubiquitination site K21 is conserved in both human and mouse HK2 (Fig. 5a and Supplementary Fig. 4a). We found that mutation on K21 slightly impaired the ubiquitination and mitochondrial localization of human HK2, albeit the effects were not as profound as what caused by the K104R or K21/104R mutation (Fig. 5a, b and Supplementary Fig. 4b, c). Previous studies and our findings collectively suggest that Akt-mediated HK2 phosphorylation is a consensus mechanism regulating mitochondrial association of both human and mouse HK2 whereas HectH9-medaited HK2 ubiquitination at K104 is selective for human HK2.

Chemoresistant tumor recurrence accounts for a large portion of cancer deaths. Mounting evidence has linked dysregulated metabolism to drug resistance^9,57–59^. For instance, hypoxia, which is known to activate glycolysis, has been found to increase the CSC population. In addition, the acidic microenvironment resulting from elevated glycolysis in tumors promotes the growth of CSCs. While several glycolytic enzymes are known to exhibit direct regulation in the growth and migration of cancer cells, their roles in CSC functions remains obscure. A recent report revealed that PKM2 is a direct CSC regulator by regulating β-catenin^60^. The current study uncovers an indispensable role of HK2 and its upstream activator HectH9 in driving CSC expansion and chemoresistance. Our research further showed that the HectH9/HK2 pathway regulates CSC self-renewal by ROS-blockade. We found that NAC increased CSC self-renewal in a dose-dependent manner and offsets the anti-CSC efficacy resulting from HectH9 or HK2 deficiency. Our observations support the previous notion that antioxidants including NAC and vitamin E accelerate the progression of lung cancer and melanoma in two animal studies^61,62^. These results collectively provide an explanation of why antioxidants might adversely affect clinical outcomes in cancer patients^63,64^, suggesting that the antioxidant supplements should be used with caution.

Our study adds HK2 to the growing list of oncogenic substrates of HectH9. HectH9 upregulation has been documented in a number of human cancers including breast, lung and colon^31^. However, HectH9’s pathological role in tumor development appears to be context-dependent. Depletion of HectH9 in human breast cancer arrests proliferation via suppression of the Myc target gene^26^. In line with these observations, Eilers and Wu’s groups demonstrated that HectH9 inhibition suppressed the growth of human colon and lung cancer in orthotopic xenograft models^27,47^. In contrast, Mak’s group showed that HectH9 decreased Myc and EphB3 expression and suppressed colon tumor development in the APC^min^ mouse model^65^. In a carcinogen-induced skin cancer mouse model, HectH9 acts as a tumor suppressor by stabilizing the Myc/Miz1 complex^66^. Further investigation is needed to clarify whether the discrete HectH9 phenotypes reported in these studies are evidence that HectH9 is a context-dependent oncoprotein or whether they arise from the different biology of human and mouse tumors. Four independent bioinformatics analyses have reported the upregulation of the HectH9 gene in human prostate cancer from different patient cohorts. In support of this notion, our histopathological studies uncovered that HectH9 protein is overexpressed in prostate tumor specimens and that such overexpression is closely correlated with disease progression. Moreover, our histological analyses showed that HK2 protein is upregulated in prostate cancer (Supplementary Fig. 7a–d and Table 1). Despite HK2 overexpression is not caused by HectH9 but likely due to the frequent loss of Pten and p53 in prostate tumors, the co-upregulation of HectH9 and HK2 implicates HectH9’s pathological relevance in glycolytic tumors. Decreased Myc protein expression appears to be a convergent mechanism underlying HectH9’s tumor suppressive function^65,66^, we thus tested if HectH9 alters Myc expression in prostate cancer. As shown in Supplementary Fig. 7e, HectH9 deficiency did not cause Myc stabilization in prostate and breast cancer cells, reducing the likelihood that the anti-cancer activities by HectH9 targeting in these cancer types would be countered by Myc upregulation. Our research together shows the promise of targeting HectH9 E3 ligase in prostate and metabolism-addicted tumors.

## Methods

### Cell culture and reagents

PC-3, MDA-MB-231, HeLa and 293 T cells (from ATCC) were cultured in DMEM containing 10% fetal bovine serum and 1% penicillin/streptomycin. The cell lines were authenticated by short tandem repeat profiling and routinely verified to be free of mycoplasma contamination by the R&D MycoProbe® Mycoplasma Detection Kit. The HA-HectH9 construct, encoding 2473–4373 residues of catalytically active HectH9, was kindly provided by Dr. M. Eilers^26^. Full-length and kinase dead mutant of GFP-tagged HK2 constructs (full-length HK2-pGFPN3 and mutant HK2-pGFPN3) were gifts from Dr. H. Ardehali (Addgene plasmids #21920 and #21922). The HA-HectH9 catalytically defective mutant (C4341A) and various HK2 lysine mutants were generated using the QuikChange site-directed mutagenesis kit (Stratagene), according to the manufacturer’s protocol. Doxorubicin, paclitaxel and 2-DG were from Sigma.

### Immunoblotting and immunoprecipitation analysis

Cells were harvested and lysed with RIPA buffer containing a protease inhibitor cocktail (Roche) for IB analysis. The following antibodies were used for IB analysis: anti-ARF-BP1 antibody (1:1000–Abcam), anti-Hexokinase 2 (1:2000–Santa Cruz), anti-Hexokinase 2 (1:1000 –Cell Signaling), anti-Hexokinase I (1:1000–Cell Signaling), anti-α-Tubulin (1:10,000–Sigma), anti-GRP78 (1:10,000–BD Sciences), anti-β-Actin (1:10,000–Sigma), anti-Flag (1:1000–Sigma), anti-GFP (1:1000–Cell Signaling), anti-Myc (1:2000–Roche), anti-HA (1:1000– Sigma), anti-p53 (1:10,000–Santa Cruz), anti-K63-Ub (1:1500–Cell Signaling), anti-K48-Ub (1:1,000–Cell Signaling) and anti-His (1:1000 –Santa Cruz). For IP, cells were lysed with E1A lysis buffer (250 mM NaCl, 50 mM HEPES, pH 7.5, 0.1% NP40, 5 mM EDTA) and a protease inhibitor cocktail (Roche). Cell lysates were immunoprecipitated with anti-HA antibody (Sigma), anti-HK2 antibody (Santa Cruz) and anti-Lasu1 (HectH9; Bethyl Lab), according to manufactory’s instructions. Detailed antibody information is listed in Supplemental Table 1. Uncropped scans for blots are presented in Supplementary Fig. 12.

### Viral infection

293T cells were seeded and allowed to attach overnight. Cells were then transfected with envelope plasmid (VSV-G), packing plasmid (deltaVPR8.9) and HectH9, HK2 or GFP shRNA using calcium phosphate transfection methods. HectH9-lentiviral shRNA-1 (5’- GCTCCCACTATAACCTCACTT-3’), HectH9-lentiviral shRNA-2 (5’-CGACGAGAACTAGCACAGAAT-3’), HK2-lentiviral shRNA-1 (5’-GCTTGAAGATTAGGTACTATC-3’), HK2-lentiviral shRNA-2 (5’-CCAAAGACATCTCAGACATTG-3’) or Myc-lentiviral shRNA (5’- CCTGAGACAGATCAGCAACAA-3’) were transfected along with the packing plasmids. Host cells were then seeded to allow for ~50% confluency for infection the next day. Forty-eight hours post-transfection, virus particles were used to infect mammalian cells. All infected cells were then cultured in medium containing the appropriate antibiotics for selection.

### ECAR measurements

PC-3 cells were seeded at 2 × 10^4^ cells/well on a Seahorse Biosciences 96-well plate and incubated for 16 h and then assayed with glycolytic stress test kit (Seahorse Biosciences, 103020–100). The cellular glycolytic rate, represented as the ECAR, was measured by using a Seahorse Biosciences XF96 analyzer.

### ^13^C tracer studies

Cells were seeded in triplicate, allowed to grow overnight and labeled with glucose or [U_6_-^13^C_6_]-glucose (>99% purity and 99% isotope enrichment for each carbon position; Cambridge Isotope Labs) to profile overall central carbon metabolism^67^. After labeling, culture medium and cell pellets were collected for extraction and analysis, respectively. Cells pellets were scraped into 50% methanol:water, snap frozen three times, spun down and the supernatant was isolated. The supernatant was then dried down and methoximated and derivatized. G-6-P was monitored at *m*/*z* 357–359, lactate at *m*/*z* 219–222, and pyruvate at *m*/*z* 174–177. Citrate was monitored at 465–471, succinate at *m*/*z* 247–251, fumarate at *m*/*z* 245–249, malate at *m*/*z* 335–339, and α-ketoglutarate at *m*/*z* 304–309. Ten nmoles of adonitol were used as an internal standard for analyzing the relative abundance of ^13^C-labeled metabolites. To monitor the lactate secreted into the media, the media was dried down and processed as described above. The IsoCor software was used to perform appropriate data correction by taking into account the contribution of naturally occurring isotopes brought by the derivatization reagent. For metabolite profiling in tumors, 1 mg/g of ^13^C-labeled glucose was intraperitoneally injected into nude mice bearing PC-3-derived tumor xenografts (~600 mm^3^ in volume). Three hours later, tumors were isolated for metabolite analysis as described above. Metabolites from tumors were normalized to protein content.

### GC-MS analysis

GC-MS spectral data were analyzed using the HP5973 mass selective detector connected to an HP6890 gas chromatograph. The settings used are as follows: GC inlet 230 °C, transfer line 280 °C, MS source 230 °C and MS Quad 150 °C. An HP-5 capillary column (30 m length, 250 µm diameter, 0.25 µm film thickness) was used to analyze lactate and intermediates of glycolytic and TCA cycles.

### Ubiquitination assay

293T cells were transfected with indicated plasmids. After 48 h, cells were harvested and lysed with denaturing buffer (6 M guanidine-HCl, 0.1 M Na_2_HPO_4_/NaH_2_PO_4_, 10 mM imidazole). The cell extracts were then incubated and rotated with nickel beads for three hours, washed and subjected to IB analysis. For in vitro ubiquitination assays, recombinant GST-HectH9 (3228–4374) proteins that are known to preserve E3 ligase activity^26^ were expressed and purified from the bacterial lysates of BL21 cells. Flag-HK2 was expressed in 293 T cells, immunoprecipitated by anti-Flag antibody, and eluted from protein A/G beads using Flag peptides according to manufacturer’s standard procedures. Purified GST-HectH9 and Flag-HK2 proteins were incubated for three hours at 37 °C in reaction buffer (20 mM HEPES at pH 7.4, 10 mM MgCl_2_, 1 mM DTT, 59 mM ubiquitin, 50 nM E1, 850 nM Ubc13/Uev1a, 1 mM ATP, 30 mM creatine phosphate and 1 U of creatine kinase). After incubation, protein mixtures were diluted in E1A buffer and the supernatant fluid was precleared with protein A/G beads for two hours, and immunoprecipitated overnight with anti-Flag antibody, after which protein A/G beads were added for an additional two hours. Beads were washed four times with E1A buffer. Proteins were eluted in SDS-sample buffer and subjected to IB analysis.

### Immunofluorescent staining

For immunostaining in PC-3 and MDA-MB-231 cells, cells were seeded on a 4-well chamber slide (Falcon) overnight, washed with PBS and fixed at −20 °C with pre-chilled methanol for 20 min. After fixation, cells were rinsed with cold acetone for three minutes and air-dried. Slides were then blocked with 10% BSA in PBS and subjected to staining with anti-VDAC (Millipore) and anti-HK2 antibodies (Santa Cruz). Alexa-594-conjugated goat anti-rabbit or Alexa-488-conjugated goat anti-mouse secondary antibodies (both from Molecular Probes) were applied prior to visualization of images using a Leica SP5 confocal microscope. For co-localization of GFP-HK2 and VDAC in HeLa cells, cells were seeded on a 4-well chamber slide (Falcon) overnight post transfection for 24 h, washed with PBS three times, then fixed with 3% Paraformaldehyde for 30 min. After fixation, cells were permeabilized with 0.5% Triton X-100 for 10 min and subjected to staining with anti-VDAC antibody, followed by Alexa-594-conjugated goat anti-rabbit secondary antibody for imaging. The HK2/VDAC co-localization was analyzed by two methods. One was to calculate the percentage of cells expressing co-localized HK2/VDAC in each experimental group. At least 86 cells were counted per group. The other was to quantify the co-localization by HK2 and VDAC by the Pearson’s correlation coefficient^68,69^. Fluorescent images were analyzed with Fiji (ImageJ, NIH). The co-localization of HK2 with VDAC was assessed by the Coloc2 function. Pearson’s correlation coefficient was calculated for individual cells, which were chosen randomly and only analyzed when they express both HK2 and VDAC. 80–102 cells were counted per group. A Pearson’s correlation coefficient value of 1 indicates a perfect positive linear correlation and a value of 0 indicates no correlation.

### Mitochondrial fractionation

Mitochondrial fractionation was performed using a Mitochondria Isolation Kit for Culture Cells (Pierce). Briefly, cells were harvested and lysed with Dounce homogenization according to the manufacturer’s instructions. The isolated mitochondrial pellets were then lysed with RIPA buffer, followed by immunoblotting.

### Lactate production assay

MDA-MB-231 and HeLa cells were seeded at 6 × 10^4^ cells/well and PC-3 cells were seeded at 4 × 10^4^ cells/well in a 24-well plate in triplicate. Cells were allowed to attach overnight before serum starvation for 24 h. Cells were then replenished with complete media for seven hours (HeLa) or nine hours (MDA-MB-231 and PC-3). Culture medium was then removed from the cells and lactate concentration (mmol/L) was determined with lactate test strips and a Nova Biomedical Lactate Plus Meter. Cells were then harvested and stained with trypan blue and only viable cells were counted using a hemocytometer under a microscope. Lactate production was determined (lactate/cell number) and normalized with the control group.

### Apoptosis assay

Cells were seeded and allowed to attach overnight in 6 cm dishes. They were then treated either with 2-DG or glucose-free media for 72 h and allowed to incubate. When ready to harvest for annexin V and propidium iodide staining, they were rinsed once with 1× PBS and trypsinized. They were then stained according to the protocol obtained from the BD Biosciences FITC-annexin V Apoptosis Detection Kit I and analyzed by flow cytometry.

### Cell viability assay

PC-3, MDA-MB-231 and HeLa cells were seeded at 3000 cells/well in a 12-well plate in triplicate and allowed to incubate overnight in complete media. When ready to be counted on their respective days, cells were washed with 1X DPBS, trypsinized and re-suspended with complete media. Trypan blue exclusion was used to stain the dead cells and the viable cells were counted using a hemocytometer under a microscope. For the glucose deprivation assay, PC-3 cells were seeded at 80,000 cells/well in 6-well plates in triplicate in complete media. After incubating overnight, cells were refreshed with complete media or glucose-free media for 48 h and trypsinized for cell counting.

### Hexokinase kinase activity assay

293T cells transfected with the desired plasmids in 10 cm dishes were harvested after 48 h and resuspended with 0.3 mL of lysis buffer (45 mM Tris HCl, pH 8.2, 50 mM KH_2_PO_4_, 10 mM glucose, 11.1 mM monothioglycerol, 0.5 mM EDTA, 0.2% Triton-X-100). Brief sonication was performed twice at 25% amplitude for 12 s each and centrifuged for 5 min at 13,000 rpm. The supernatant was then collected for hexokinase assay, as well as immunoblotting. A total of 195 μL of the reaction system (0.1 M TEA buffer, 65 mM MgCl_2_, 1.1 M glucose, 27 mM adenosine 5’-triphosphate, 8.3 mM NAD, 24 U/mL G6P-DH) and 5 μL of the cell lysate were pipetted into each well of a 96-well plate in triplicate. Values were obtained at a wavelength of A_340_ using a spectrophotometer. To calculate the final hexokinase activity fold change, the value obtained at 30 min was subtracted from the baseline value and normalized to the protein concentration and the vector alone control.

### Measurement of ROS production

GFP-knockdown and HectH9-knockdown PC-3 cells were seeded and allowed to attach overnight in 60 mm dishes. Cells were then incubated with 5 μM CellROX Green Reagents (Invitrogen) for 30 min at 37 °C and subjected to flow cytometry for measuring ROS production according to the manufacturer’s instructions.

### Quantification and sorting of CD44^+^/ALDH^+^ and ALDH^+^ cells

The population of CD44^+^/ALDH^+^ and ALDH + cells were quantified or sorted by flow cytometry. For the staining of CD44^+^, cells were incubated with blocking reagent FcR (Miltenyi Biotec) for 20 min at 4 ℃ and then stained with PE conjugated anti-human CD44 antibody (BD Pharmingen), according to the manufacturer’s protocol. ALDH + cells represents the cell population exhibiting high aldehyde dehydrogenase (ALDH) activity. ALDH enzyme activity was determined by ALDEFLUOR kit (STEMCELL Technologies). Briefly, cells were washed with PBS and suspended in assay buffer to a concentration of 1 × 10^6^ cells/mL, then mixed with ALDH substrate and incubated for 40 min at 37 ℃. Diethylaminobenzaldehyde (DEAB), an ALDH-specific inhibitor, was used as negative control.

### Tumor sphere formation assay

Various cancer cells were seeded in 6-well ultra-low attachment plates (Corning) in plating medium (MEGM) for the formation of non-adherent spheroids, termed tumor spheres, which have the ability to self-renew^70^. The appearance of tumor spheres for PC-3 and HeLa cells were evaluated after 14 and 8 days, respectively. Tumor spheres with diameters ≥ 100 μm were manually counted under a microscope. Spherical diameters were calculated under light microscope using NIS-Element software (Nikon).

### Animal studies

For the xenograft tumor mouse model, 5 × 10^6^ of PC-3 cells with Luciferase or HectH9 knockdown were injected subcutaneously into the right flanks of athymic nude mice (NCr-Foxn1^nu^) purchased from Envigo. A total of 5 × 10^6^ of MDA-MB-231 or HeLa cells expressing vector control, WT or the K21/K104R mutant of HK2 were injected subcutaneously into the right flanks of athymic nude mice from Envigo. Tumor size was measured weekly with a caliper, and tumor volume was calculated by using the standard formula L × W^2 ^× 0.52, where L and W are the length and width, respectively. Mice were between 6 to 8-weeks-old at the time of experiments. Male mice were used for inoculation of PC3 cells while female mice were used for inoculation of HeLa and MDA-MB-231 cells. Mice were randomly grouped for tumor cell inoculation. Blinding was not done during tumor inoculation but during the measurement of tumor size. All experimental procedures strictly complied with the IACUC guidelines and were approved by the IACUC of Stony Brook University (IACUC-approved protocols #984737). All animal studies were complied with relevant ethical regulations for animal testing and research.

### Immunohistochemistry and scoring

Sections 3 mm in thickness were cut onto adhesive-coated glass slides. For staining of human samples, the slides were incubated with primary antibodies targeting HectH9 (1:200–Abcam) and HK2 (1:50–Cell Signaling). Primary antibodies were detected using the ChemMate DAKO EnVision kit (DAKO, K5001). The slides were incubated with the secondary antibody for 30 min and developed with 3,3’-diaminobenzidine for 5 min. Incubation without the primary antibody was used as a negative control. Immunoexpression was scored by two pathologists using a multi-headed microscope to reach a consensus for each case. The staining was evaluated based on a combination of both the percentage and intensity of positively stained tumor cells to generate an H-score, which was calculated using the following equation: H-score = ΣPi (i + 1), where i is the intensity of the stained tumor cells (0 to 4+), and Pi is the percentage of stained tumor cells for each intensity. Patient tissues used in this study were obtained with consent under the protocol approved by the institutional review board (IRB # 10501–007) of the Chi-Mei Foundational Medical Center.

### Statistical analysis

Data are shown as the means ± SD or the mean ± SEM for three independent experiments, unless otherwise indicated. All statistical significance was determined by two-tailed Student’s *t* tests, and *p* values less than 0.05 were considered statistically significant. For in vivo mouse experiments, the mouse number was determined by power analysis based on the fact that with a sample size of five and with a two-sided type I error rate of 0.05, the study would have 90% power to detect a 40% difference. For human prostate samples, the Mann–Whitney *U* test was used to assess the differential expression level of HectH9 in relation to HK2 expression status. The Spearman’s rank correlation coefficient was used to clarify the association between HectH9 expression to clinicopathological variables and HK2 expression levels. Blinding in in vivo experiments was not done during experimentation, but during outcome assessment, i.e., scoring of IHC staining of human tumor tissues and measurement of tumor size in animal studies.

### Reporting summary

Further information on research design is available in the Nature Research Reporting Summary linked to this article.

## Supplementary information

Supplementary Information

Reporting Summary

## Data Availability

Data generated from this study are included in this article and its Supplementary Information files or will be provided from the corresponding author upon reasonable request. Uncropped scans for blots are presented in Supplementary Fig. 12.
