# Supplementary material for: Non-proteolytic ubiquitination of Hexokinase 2 by HectH9 controls tumor metabolism and cancer stem cell expansion
Source: Nat Commun. 2019 Jun 14;10:2625. doi: 10.1038/s41467-019-10374-y (PMC6573064; doi:10.1038/s41467-019-10374-y)
Supplement: Supplementary file 1 — Supplementary Information [file 41467_2019_10374_MOESM1_ESM.pdf]

Supplementary Information

Non-proteolytic ubiquitination of Hexokinase 2 by HectH9 controls tumor metabolism and cancer stem cell expansion

Lee et al.

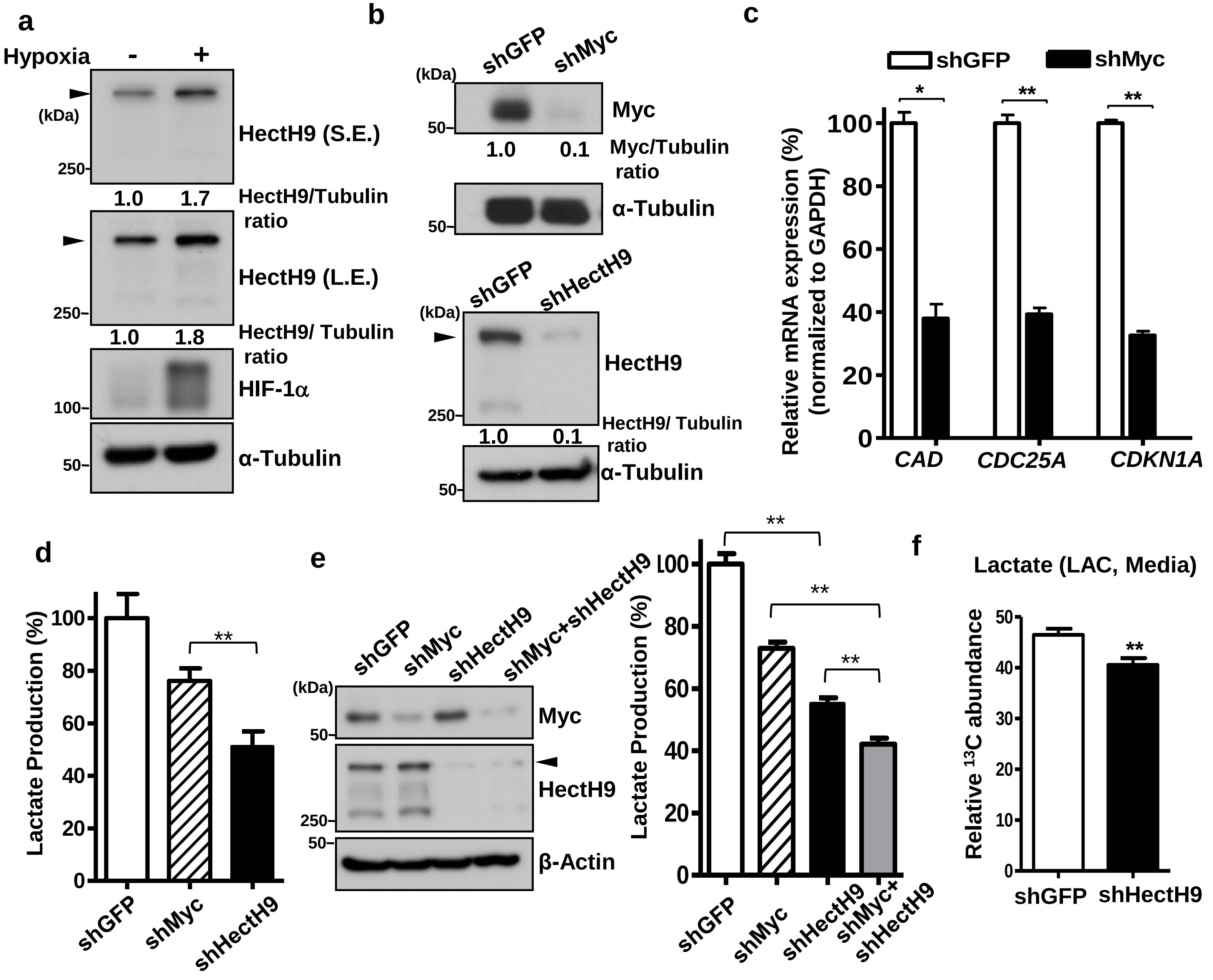

Supplementary Figure 1. Prostate cancer-associated HectH9 regulates glucose metabolism.

(a) IB analysis of HectH9 expression in PC-3 cells under normoxic and hypoxic conditions. L.E. and S.E. stand for long and short exposure, respectively. The relative intensity of protein expression as indicated was quantified with ImageJ software and normalized to α-Tubulin expression. (b) IB analysis of Myc and HectH9 expressions in PC-3 cells infected with lentiviruses containing shRNA targeting GFP, Myc or HectH9. The relative intensity of protein expression as indicated was quantified with ImageJ software and normalized to α-Tubulin expression. (c) Real-time PCR analysis for expression of the Myc-target genes including *CAD*, *CDC25* and *CDKN1A* in PC-3 cells infected with lentiviruses containing shRNA targeting GFP or Myc (n=3). (d) Lactate production assay in PC-3 cells infected with lentiviruses containing shRNA targeting GFP, Myc or HectH9 (n=3). The experiment was independently performed three times. (e) Lactate production and Western blot assays in PC-3 cells transiently infected with lentiviruses that mediate the genetic knockdown of GFP, Myc, HectH9 and Myc plus HectH9 (n=3). (f) PC-3 cells stably infected with viruses containing shRNA targeting GFP or HectH9 were labeled with [U6-$^{13}C_6$]-glucose for tracing glucose-derived metabolites. Relative abundances of ^{13}C incorporation into secreted lactate were determined by GC-MS (n=3). Results are presented as mean value ±SD; $*p<0.05$, $**p<0.01$, by Student's *t*-test. Experiments were performed at least twice. Immunoblots were performed three times.

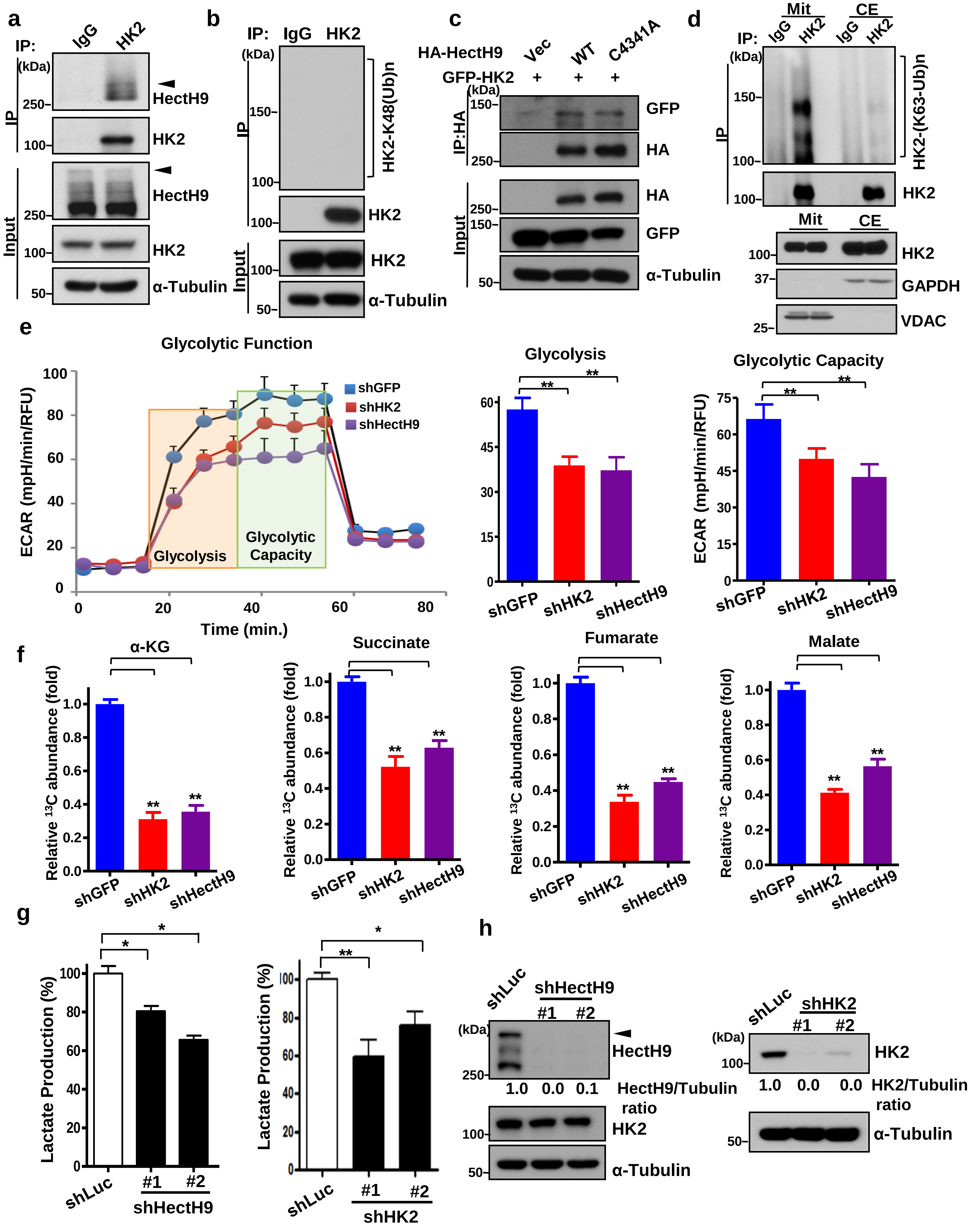

Supplementary Fig. 2

Supplementary Figure 2. HectH9 recapitulates the effects of HK2 in glucose metabolism.

(a) IP assay for endogenous HK2 and HectH9 interaction in PC-3 cells. Cell lysates were harvested for IP with IgG or anti-HK2 antibody, followed by IB analysis with indicated antibodies. (b) IP assay for measuring K48-linked ubiquitinated HK2 in PC-3 cells. Cell lysates were harvested for IP with IgG or anti-HK2 antibody, followed by IB analysis with indicated antibodies. (c) IP assay for HK2 and HectH9 interaction in 293T cells transfected with GFP-HK2 with vector alone (vec), catalytically active form (WT) or defective mutant (C4341A) of HA-HectH9. (d) IP assay for measuring the K63-linked ubiquitination of mitochondrial and cytosolic HK2 in PC-3 cells (*top*). IB assay for demonstrating the successful subcellular fractionation in PC-3 cells. VDAC is a well-established marker of mitochondrial fraction and GAPDH serves as a cytoplasmic marker (*bottom*). (e) The extracellular acidification rate (ECAR), was measured by using a Seahorse Bioanalyzer in PC-3 cells stably expressing shRNA targeting GFP, HectH9 or HK2 (n=18). Quantitative results for glycolysis and glycolytic capacity indicated by orange and green boxes are graphed in the right panel. The experiments were performed two times. (f) PC-3 cells stably expressing shRNA targeting GFP, HK2 or HectH9 were incubated with ^{13}C-labeled glucose, followed by GC/MS for measuring the abundance of glucose-derived α-KG, succinate, fumarate and malate (n=4). The relatively abundance of labeled metabolites was normalized to that in GFP-knockdown cells. (g and h) Lactate production (g) and IB (h) assays in HeLa cells infected with lentiviruses containing shRNA targeting Luciferase, HectH9 or HK2. Two HectH9 and two HK2 lentiviral shRNAs were used in this assay. The relative intensity of protein expression as indicated was quantified with ImageJ software and normalized to α-Tubulin expression. Results in (e)-(g) are presented as mean value ±SD; *p<0.05, **p<0.01, by Student's t-test. Experiments were performed at least twice in triplicates. Immunoblots were performed three times.

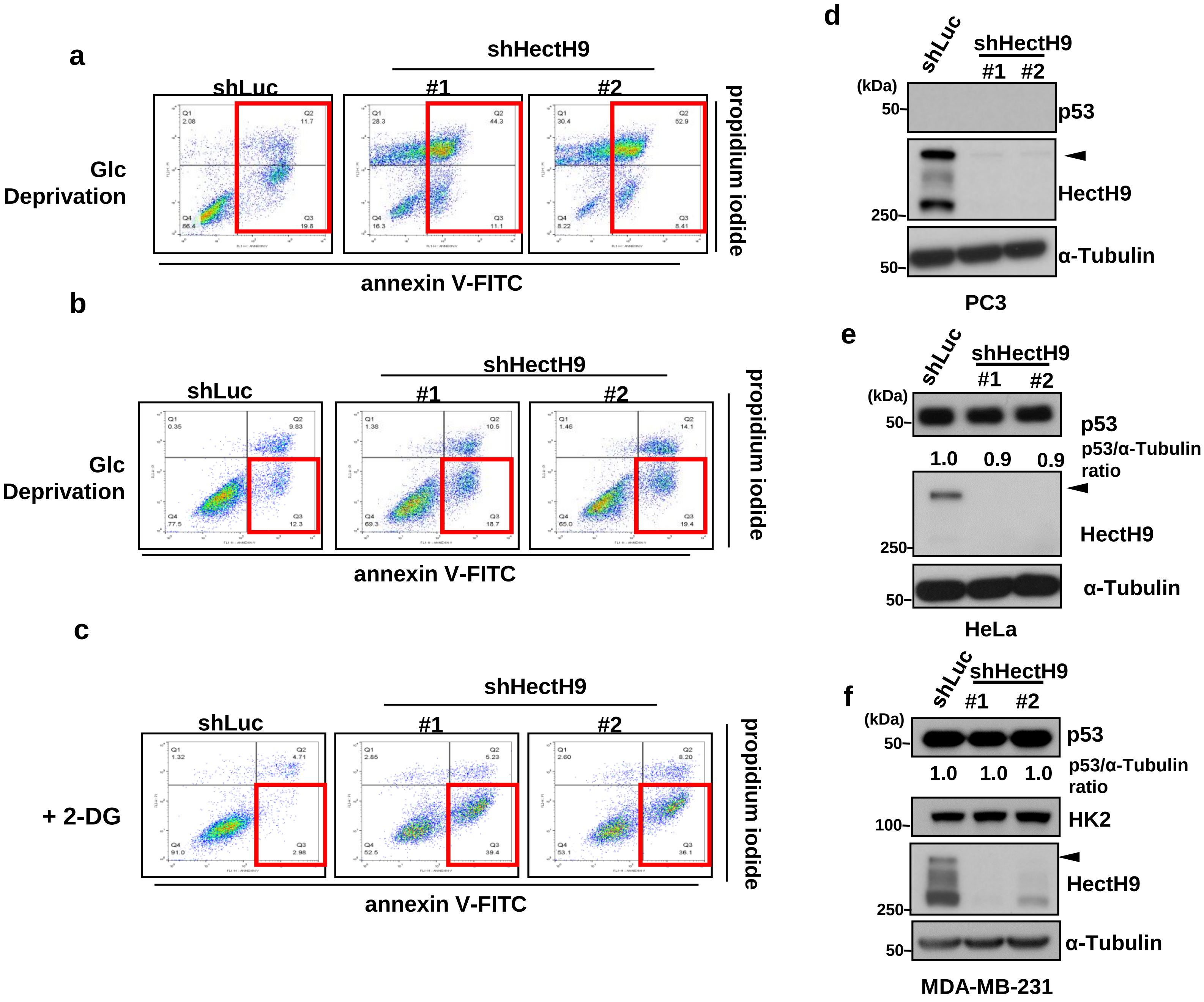

Supplementary Figure 3. HectH9 depletion promotes cancer cell apoptosis under energy stress.

(a) Representative flow cytometry analysis image of annexin V and PI staining in PC-3 cells infected with lentiviruses containing shRNA targeting Luciferase or HectH9 in the presence of glucose (Glc)-deprived media for 48 hours. (b) Representative flow cytometry analysis image of annexin V and PI staining in MDA-MB-231 cells infected viruses containing shRNA targeting Luciferase or HectH9 in the presence of Glc-deprived media for 72 hours. (c) Representative flow cytometry analysis image of annexin V and PI staining in PC-3 cells stably expressing shRNA targeting Luciferase or HectH9 in the presence of 2-DG for 72 hours. (d and e) IB analysis of p53 and HectH9 protein levels in PC-3 (d) and HeLa cells (e) infected with viruses containing shRNA targeting Luciferase or HectH9. (f) IB analysis of p53, HK2 and HectH9 protein expression in MDA-MB-231 cells infected with viruses containing shRNA targeting Luciferase or HectH9. Two HectH9 shRNAs were used in the assays shown in (a)-(f). The relative intensity of protein expression as indicated in (e) and (f) was quantified with ImageJ software and normalized to α-Tubulin expression. Experiments were repeated three times. Immunoblots were performed three times.

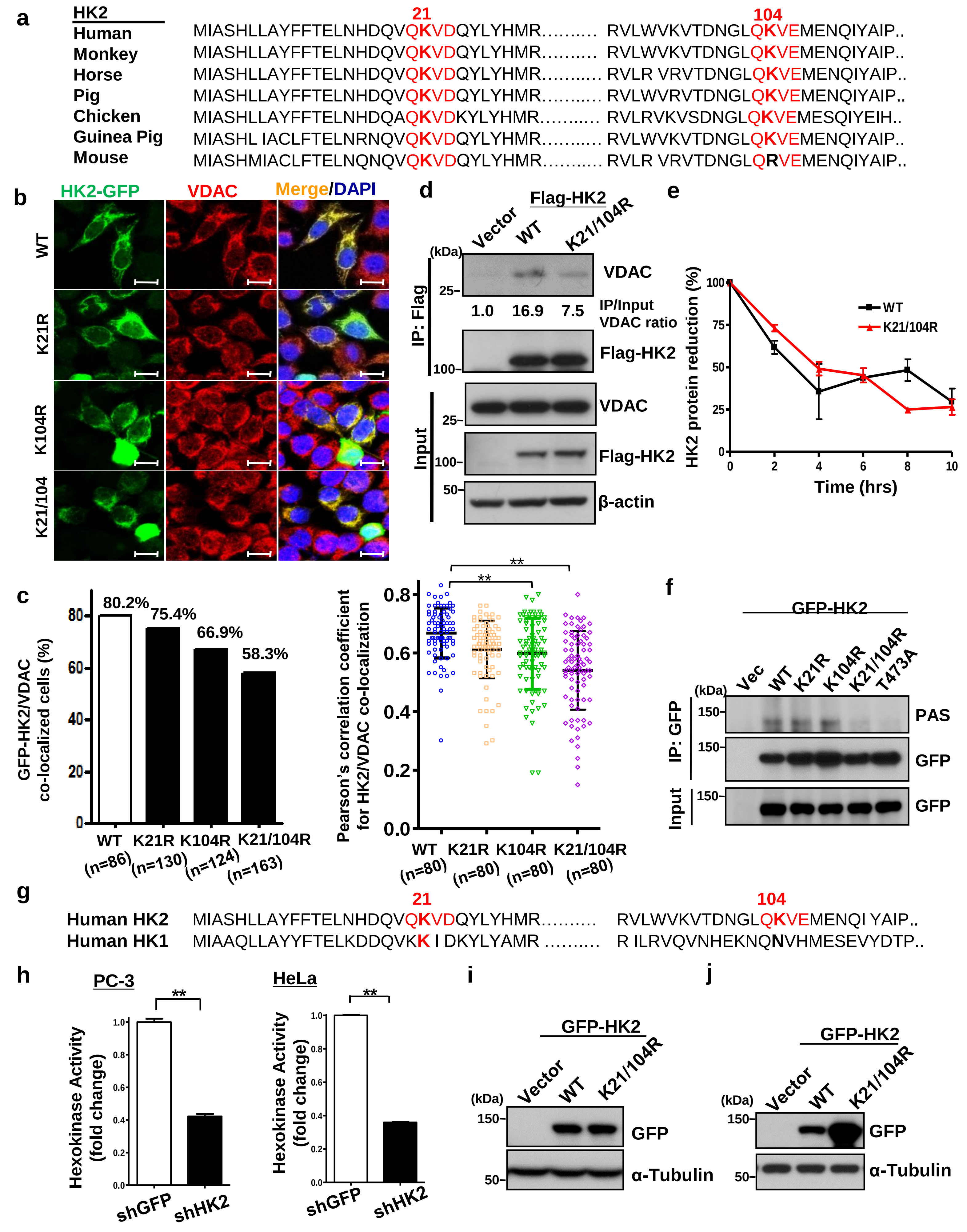

Supplementary Fig. 4

Supplementary Figure 4. K63-linked ubiquitination regulates mitochondrial localization of HK2.

(a) Identification of the lysine (K) residues on HK2 where K63-linked ubiquitination takes place. A sequence alignment revealed that the K21 and K104 residues of HK2 are evolutionarily conserved (labeled in red) among different species. (b) HeLa cells transfected with indicated plasmids were fixed and subjected for immunofluorescence staining with anti-VDAC antibody and DAPI. Scale bar represents 10 μm. (c) The left panel shows the quantification results of the percentage of cells containing HK2/VDAC co-localization in HeLa cells transfected with indicated plasmids. At least 86 cells were counted in each group from two biological replicates. The right panel shows that the HK2/VDAC correlation was determined using the Pearson's correlation coefficient. 80 cells were counted in each group from two biological replicates. (d) IP for HK2/VDAC interaction in HeLa cells transfected with indicated plasmids. The IP products and whole cell lysates (input) were harvested for IB analysis with indicated antibodies. The ratio of immunoprecipitated VDAC to total VDAC levels in the whole cell lysates was quantified using ImageJ software. (e) Cycloheximide chase assay for HK2 protein turnover in MDA-MB-231 cells stably expressing WT or the K21/104R mutant of Flag-HK2. Two independent experiments were carried out for the quantitative data as shown. (f) IP assay for HK2 phosphorylation by Akt. HeLa cells transfected with indicated constructs were harvested for IP with an anti-GFP antibody, followed by IB with a phospho-Akt substrate (PAS) antibody. The HK2 T473A mutant serves as a negative control. (g) A sequence alignment revealed that the K21, but not the K104, site is conserved (labeled in red) between human HK2 and HK1 isoforms. (h) HK activity assay in PC-3 and HeLa cells with GFP or HK2 knockdown (n=3). (i and j) IB analysis of GFP-HK2 expression in MDA-MB-231 (i) and HeLa cells (j) with HK2 knockdown, followed by ectopic expression of vector, WT or the K21/104R mutant of GFP-HK2. Experiments were performed at least twice. Immunoblots were performed three times. Results in (c) and (h) are presented as mean value ±SD; ** $p<0.01$, by Student's *t*-test.

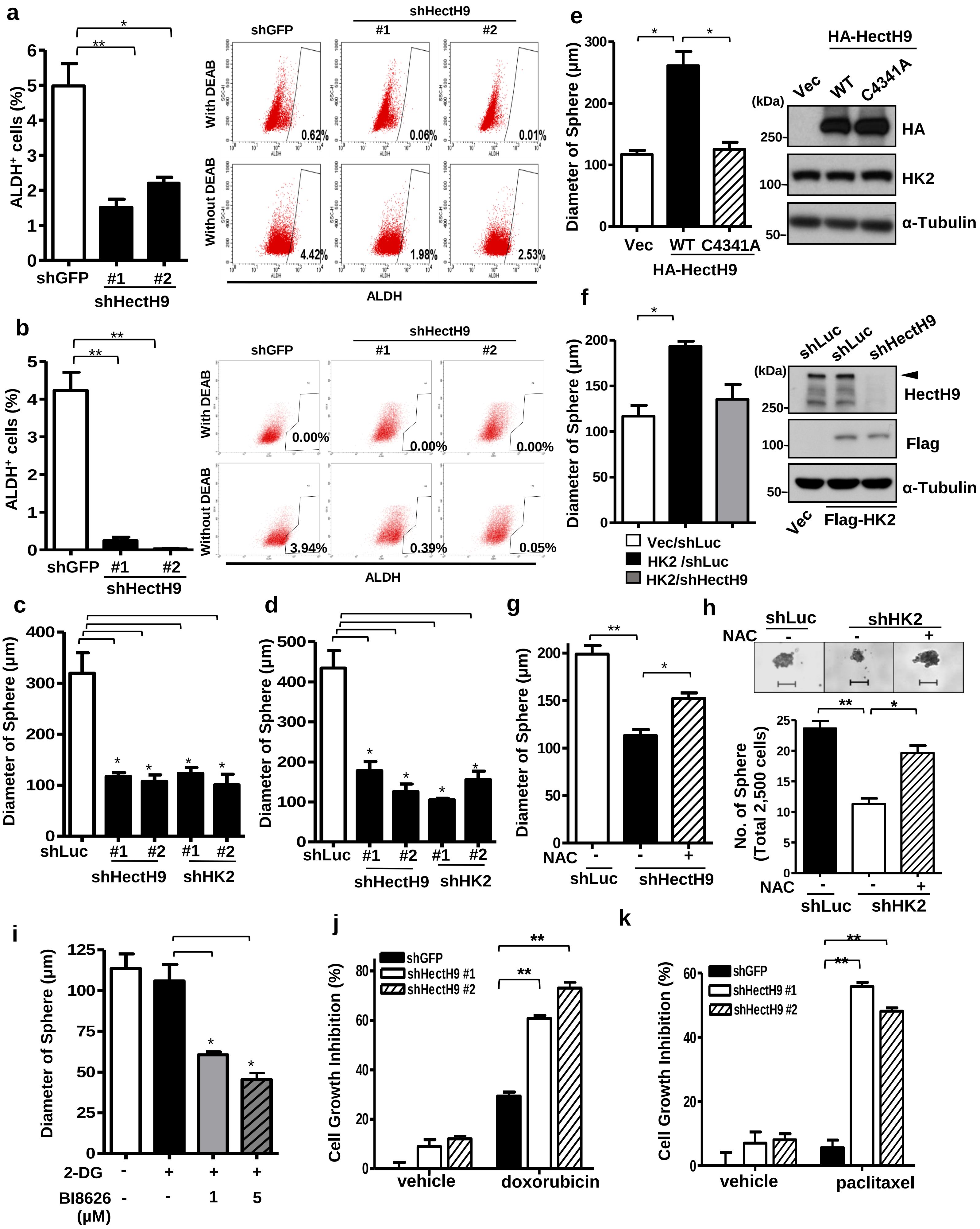

Supplementary Fig. 5

Supplementary Figure 5. HectH9 deficiency suppresses CSCs and enhances cancer cell response to chemotherapy.

(a) Population of ALDH+ cells was determined by flow cytometry analysis in HeLa cells with GFP or HectH9 knockdown (n=3). Representative flow cytometry analysis images of ALDH+ cell are shown in the right panel. Diethylaminobenzaldehyde (DEAB) was used as negative control. (b) Population of ALDH+ cells was determined by flow cytometry analysis in MDA-MB-231 cells with GFP or HectH9 knockdown (n=3). Representative flow cytometry analysis images of ALDH+ cell are shown in the right panel. DEAB was used as negative control. (c, d) Quantitative measurement of spherical diameters in PC-3 (c) and HeLa (d) cells with Luciferase or HectH9 knockdown (n=3). Diameter of spheres was calculated under light microscope using NIS-Element software (Nikon). (e) Quantitative measurement of spherical diameters and IB analysis for HA-HectH9 and HK2 protein expression in HectH9-knockdown HeLa cells transfected with vector control, catalytically active (WT) form or catalytically defective mutant (C4341A) of HA-HectH9 (n=3). (f) Quantitative measurement of spherical diameters and IB analysis for HectH9 and HK2 expression in HeLa cells that overexpress vector control or Flag-HK2, followed by Luciferase or HectH9 knockdown (n=3). (g) Quantitative measurement of spherical diameters in PC-3 cells with Luciferase or HectH9 knockdown in the absence and presence of NAC (n=3). (h) Tumor sphere formation assay in PC-3 cells with Luciferase or HK2 knockdown in the absence and presence of NAC (n=3). Scale bar represents 100 μm. (i) Quantitative measurement of spherical diameters in PC-3 cells incubated with vehicle, 2-DG alone or 2-DG and BI8626 in combination (n=3). (j and k) Cell growth inhibition assay in MDA-MB-231 cells with GFP or HectH9 knockdown in the absence and presence of doxorubicin for 96 hours (j) or paclitaxel for 48 hours (k). Cell numbers were counted by using a hemocytometer (n=3). Two HectH9 lentiviral shRNAs were used in the assays shown in (a)-(d), (j) and (k). Results are presented as mean value ±SD; $*p<0.05$; $**p<0.01$, by Student's *t*-test. All the experiments were performed at least twice in triplicates. Immunoblots were performed twice.

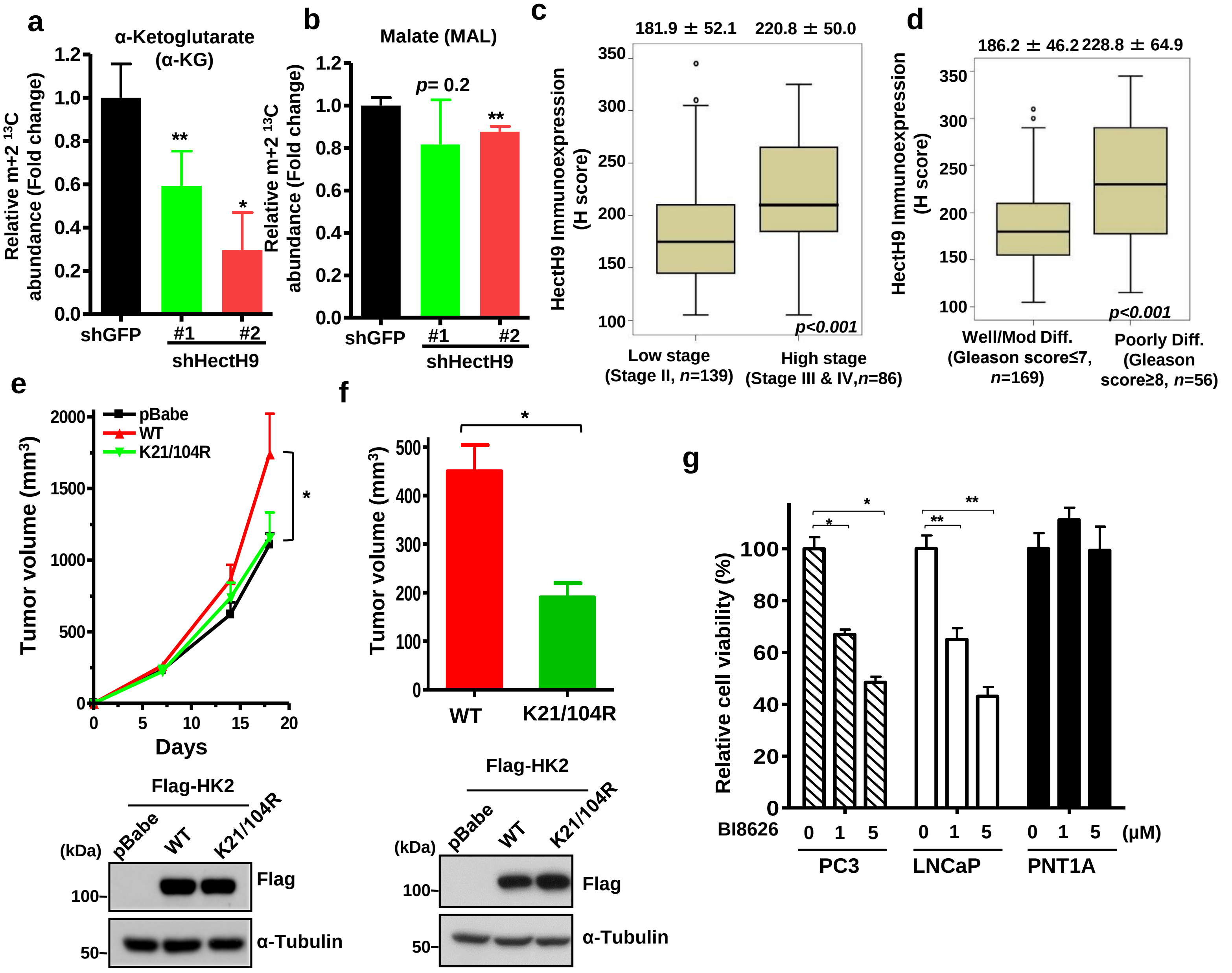

Supplementary Figure 6. HectH9 upregulation is associated with prostate cancer progression and HectH9-mediated HK2 ubiquitination regulates tumor growth.

(a and b) Xenograft tumors derived from PC-3 infected with viruses containing shRNA targeting GFP or HectH9. Two HectH9 shRNAs were used. The xenograft tumors were labeled with [U6-$^{13}C_6$]-glucose for 3 hours. Relative abundances of ^{13}C incorporation into glucose metabolites for m+2 α-ketoglutarate (a) and malate (b) in tumors (n=5) were determined by GC-MS. (c and d) Quantification analysis of HectH9 protein expressions in 225 cases of resected prostate tumors in relation to different tumor stages (c) and various differentiation statuses (d), as determined by Gleason score. (e) *In vivo* primary tumor growth derived from HeLa cells infected virus expressing vector alone (pBabe), WT or K21/104R of Flag-HK2. Cells were injected into nude mice and tumorigenesis was monitored. Quantitative results of tumor volumes are shown in the upper panel (n=7 in each group). IB analysis of Flag-HK2 expression in HeLa cells infected with viruses expressing indicated constructs is shown in the lower panel. (f) *In vivo* primary tumor growth in MDA-MB-231 cells infected viruses expressing WT or ubiquitination-defective mutant (K21/104R) of Flag-HK2. Cells were injected into nude mice and tumorigenesis was monitored. Quantitative results of tumor volumes are shown in the upper (n=6 in each group). IB analysis of Flag-HK2 expression in MDA-MB-231 cells infected with viruses expressing indicated constructs is shown in the lower. (g) Cell viability assay in PC-3, LNCaP and PNT1A cells incubated with various doses of the HectH9 inhibitor BI8626 for four days. Cell numbers were counted by a hemocytometer (n=3). Results in (a), (b), (e)-(g) are presented as mean value ±SD; $*p<0.05$, $**p<0.01$, by Student's *t*-test. Experiments were performed at least twice in triplicates. Immunoblots were performed twice.

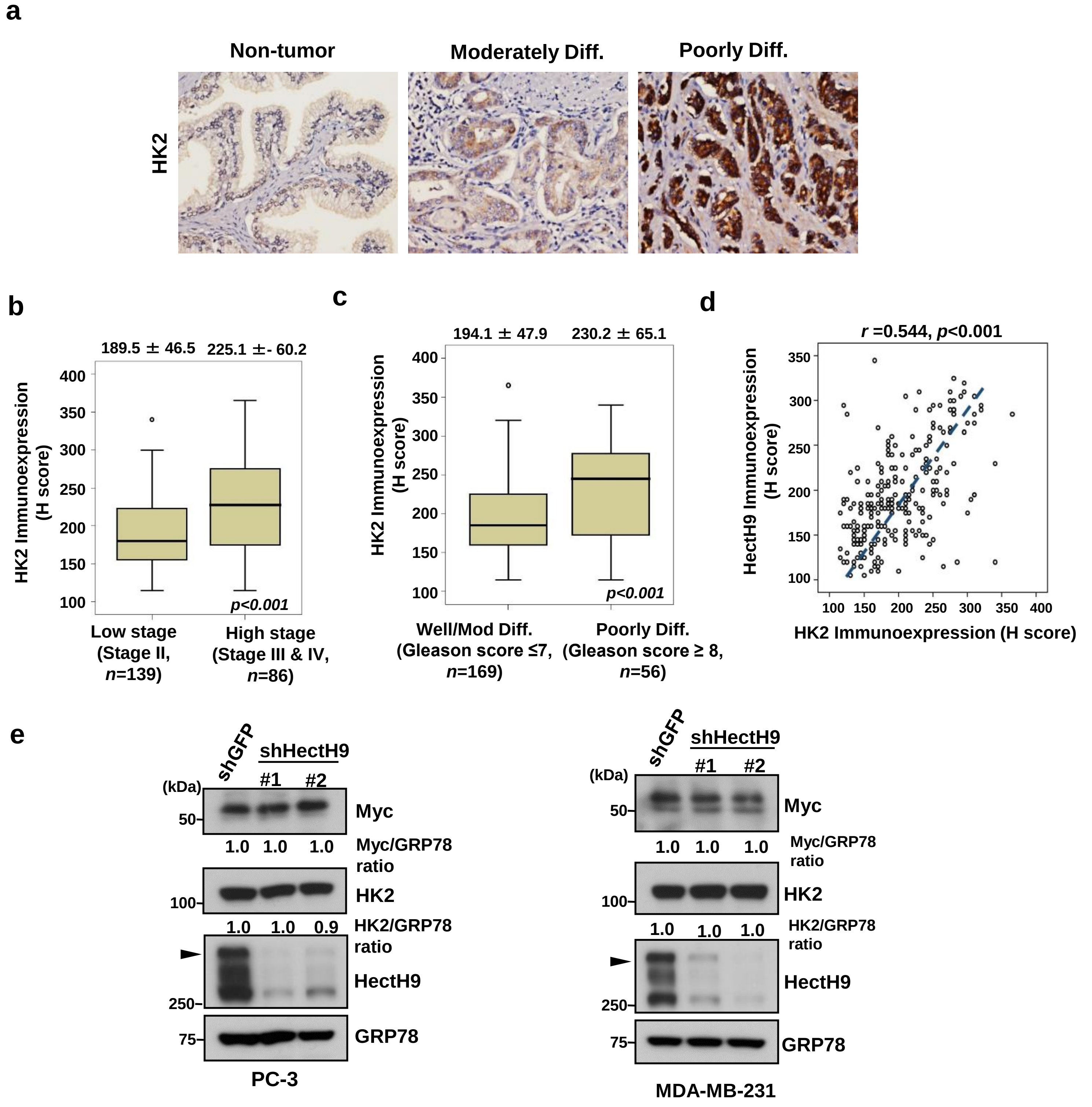

Supplementary Figure 7. HK2 expression is associated with HectH9 expression and prostate cancer progression in patients.

(a) Representative images of consecutive tissue sections for histological analyses of HK2 expressions in normal tissues and prostate tumors with moderately or poorly differentiation status. (b and c) Quantification analysis of HK2 protein expressions in 225 cases of resected prostate tumors in relation to different tumor stages (b) and various differentiation statuses (c), as determined by Gleason score. (d) Scatter plot analysis for the correlation between HectH9 expression and HK2 expression levels. The correlation was determined using the Pearson correlation coefficient (n=225). LI represents labeling index. (e) IB analysis of Myc and HK2 protein expression in PC-3 and MDA-MB-231 cells infected with viruses expressing shRNA targeting GFP or HectH9. Two different HectH9 shRNAs were used in this assay. The relative intensity of protein expression as indicated was quantified with ImageJ software and normalized to an internal control GRP78. Immunoblots were performed three times.

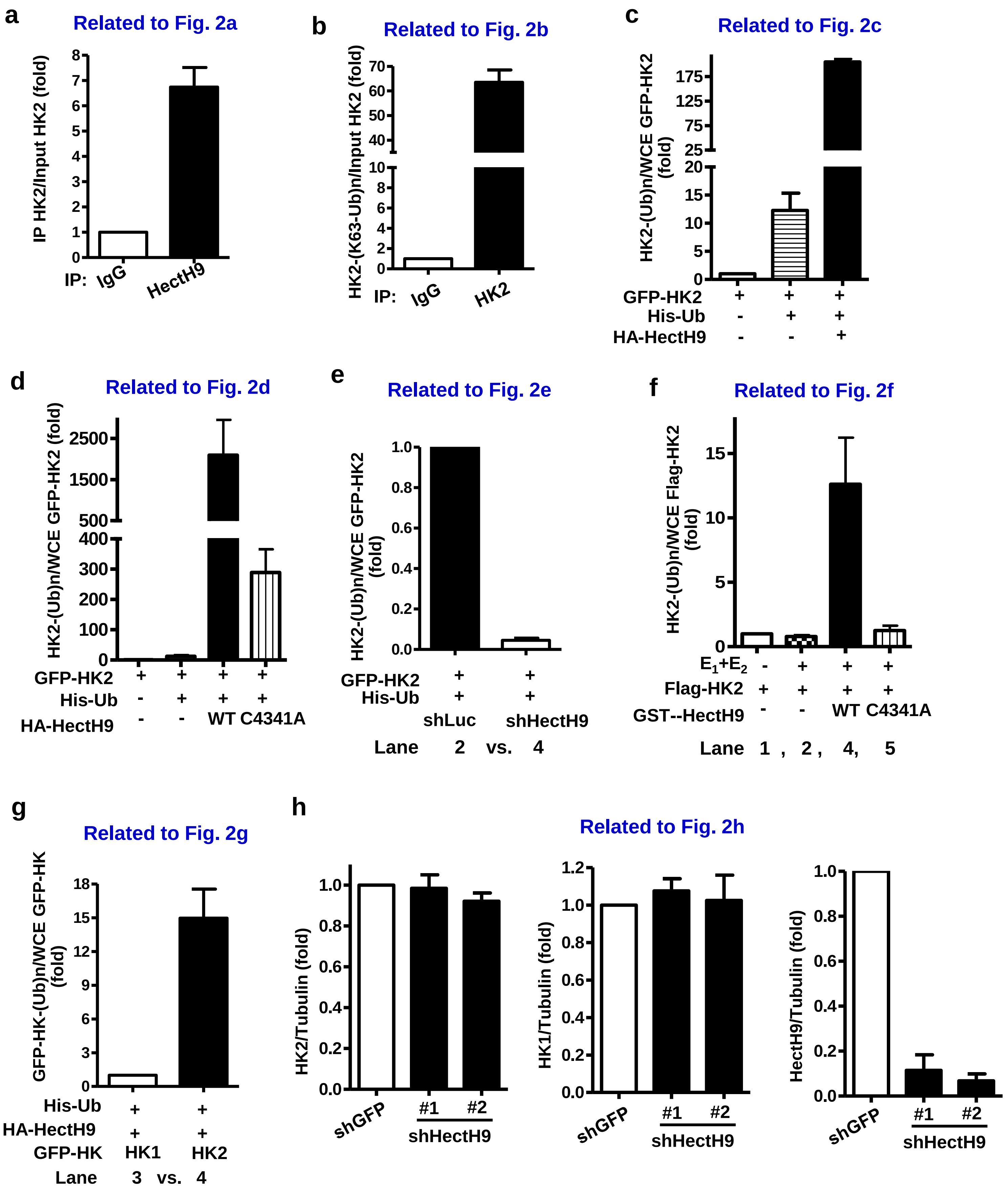

Supplementary Figure 8. Quantification results for immunoblots in Fig. 2.

The relative intensity or ratio of protein expression was quantified with ImageJ software and normalized to the indicated protein. Immunoblots were performed three times. Quantification results of repeated blots are presented as mean ± SD.

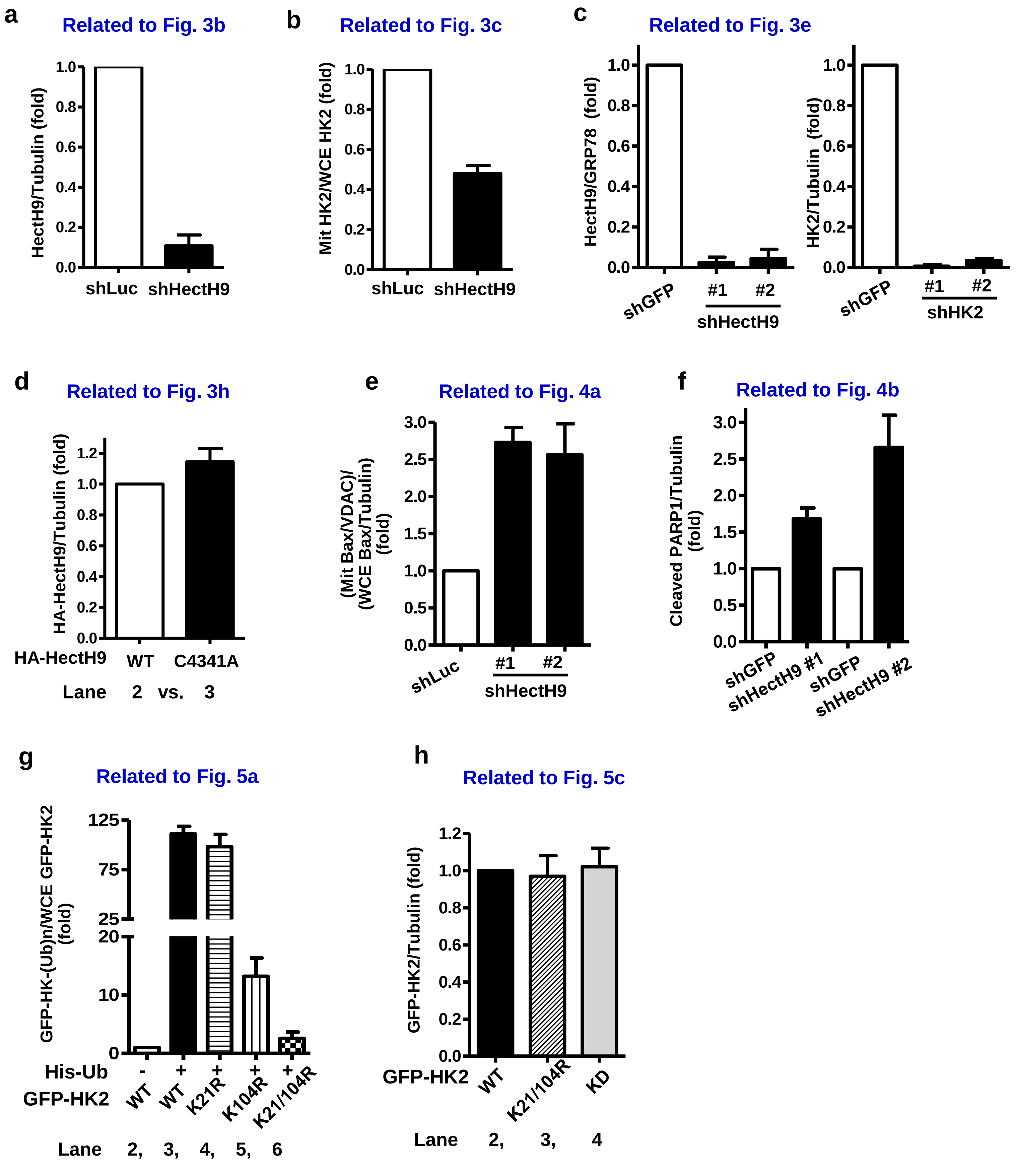

Supplementary Figure 9. Quantification results for immunoblots in Fig. 3-5.

The relative intensity or ratio of protein expression was quantified with ImageJ software and normalized to the indicated protein. Immunoblots were performed three times unless otherwise indicated. Quantification results of repeated blots are presented as mean ± SD.

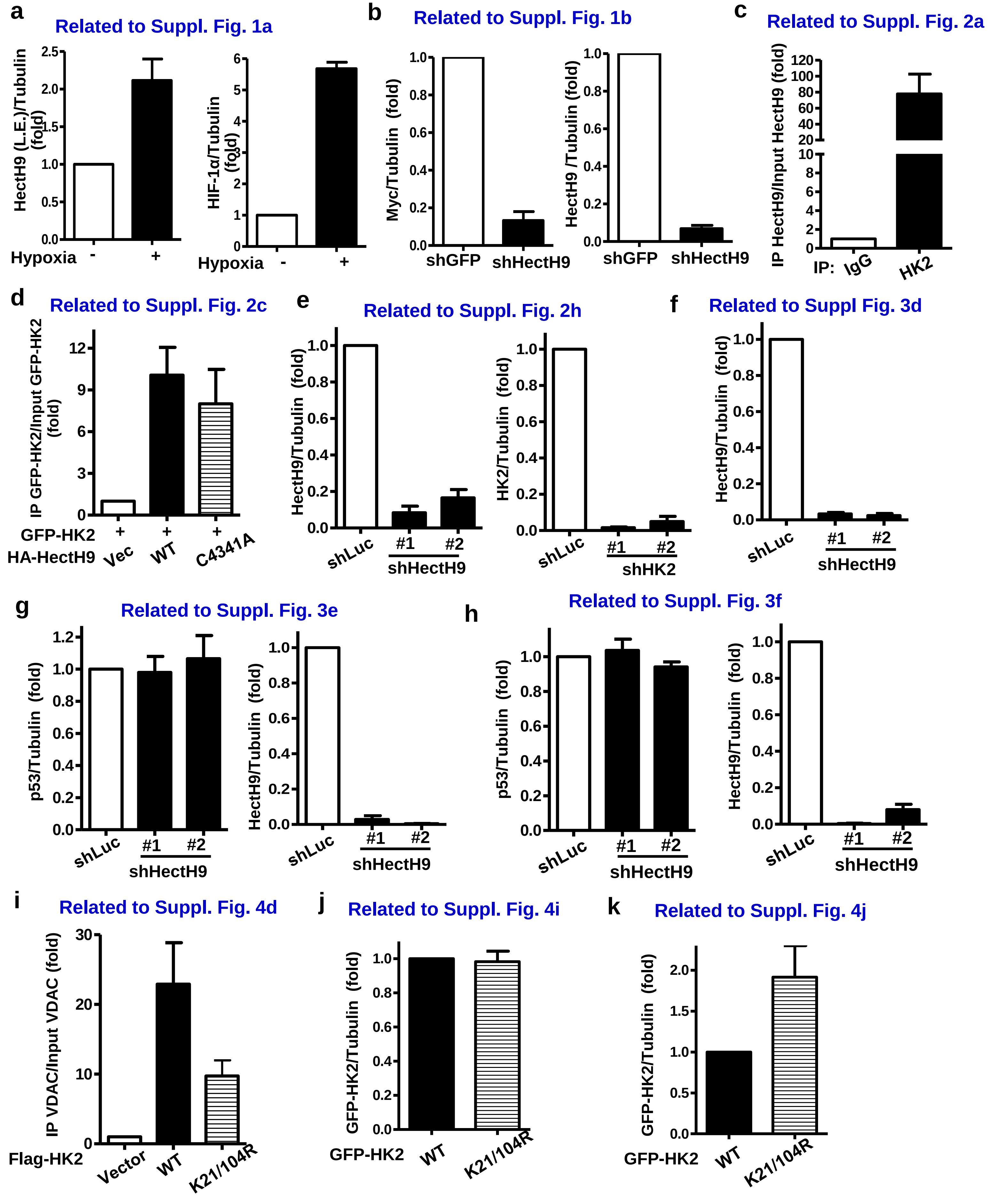

Supplementary Figure 10. Quantification results for immunoblots in Supplementary Fig. 1-4.

The relative intensity or ratio of protein expression was quantified with ImageJ software and normalized to the indicated protein. Immunoblots were performed three times. Quantification results of repeated blots are presented as mean ± SD.

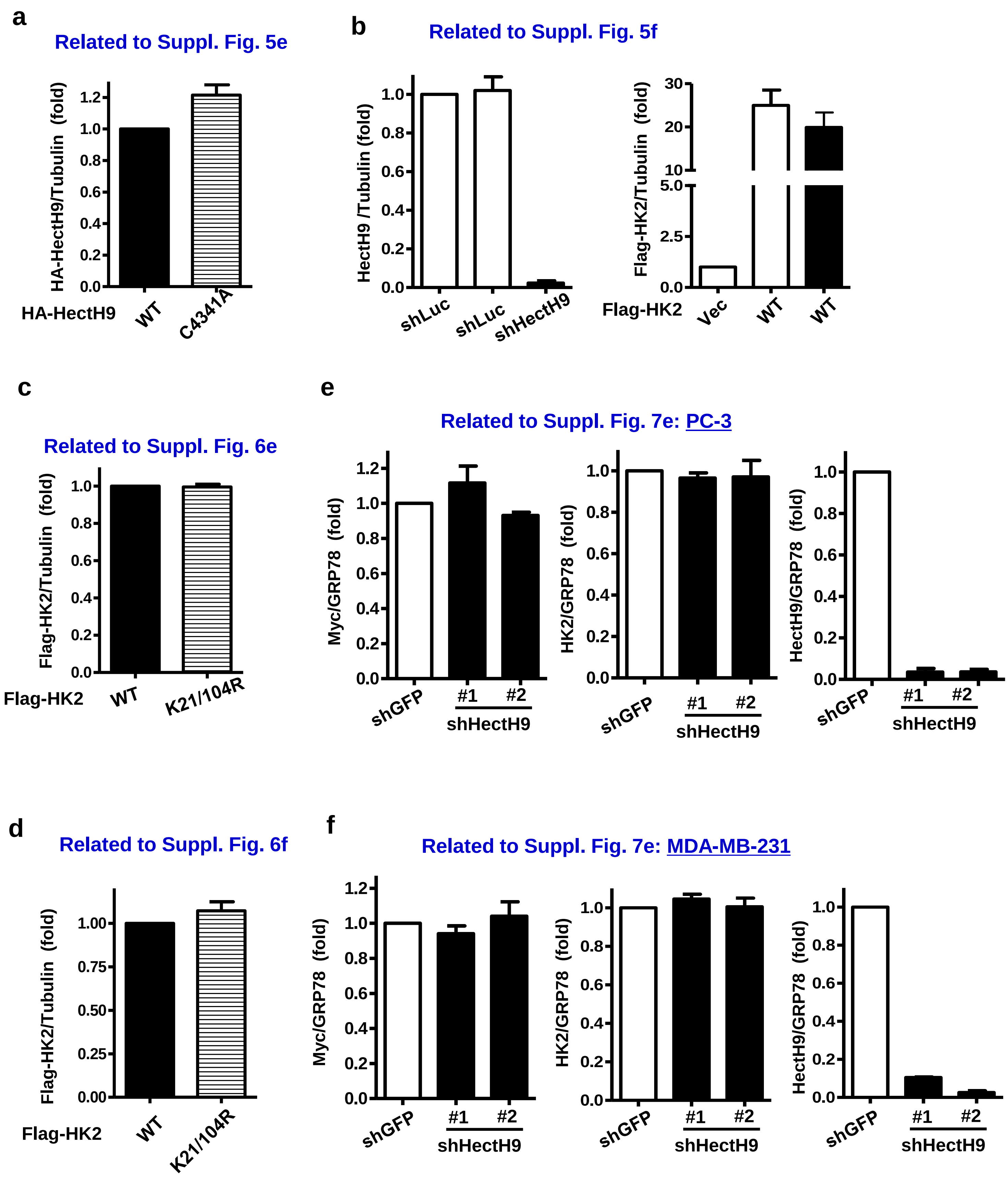

Supplementary Figure 11. Quantification results for immunoblots in Supplementary Fig. 5-7.

The relative intensity of protein expression was quantified with ImageJ software and normalized to protein as indicated. Immunoblots were performed three times unless otherwise indicated in the legends for Supplementary Fig. 5-7. Quantification results of repeated blots are presented as mean ± SD.

Supplementary Figure 12. Uncropped scants of western blots

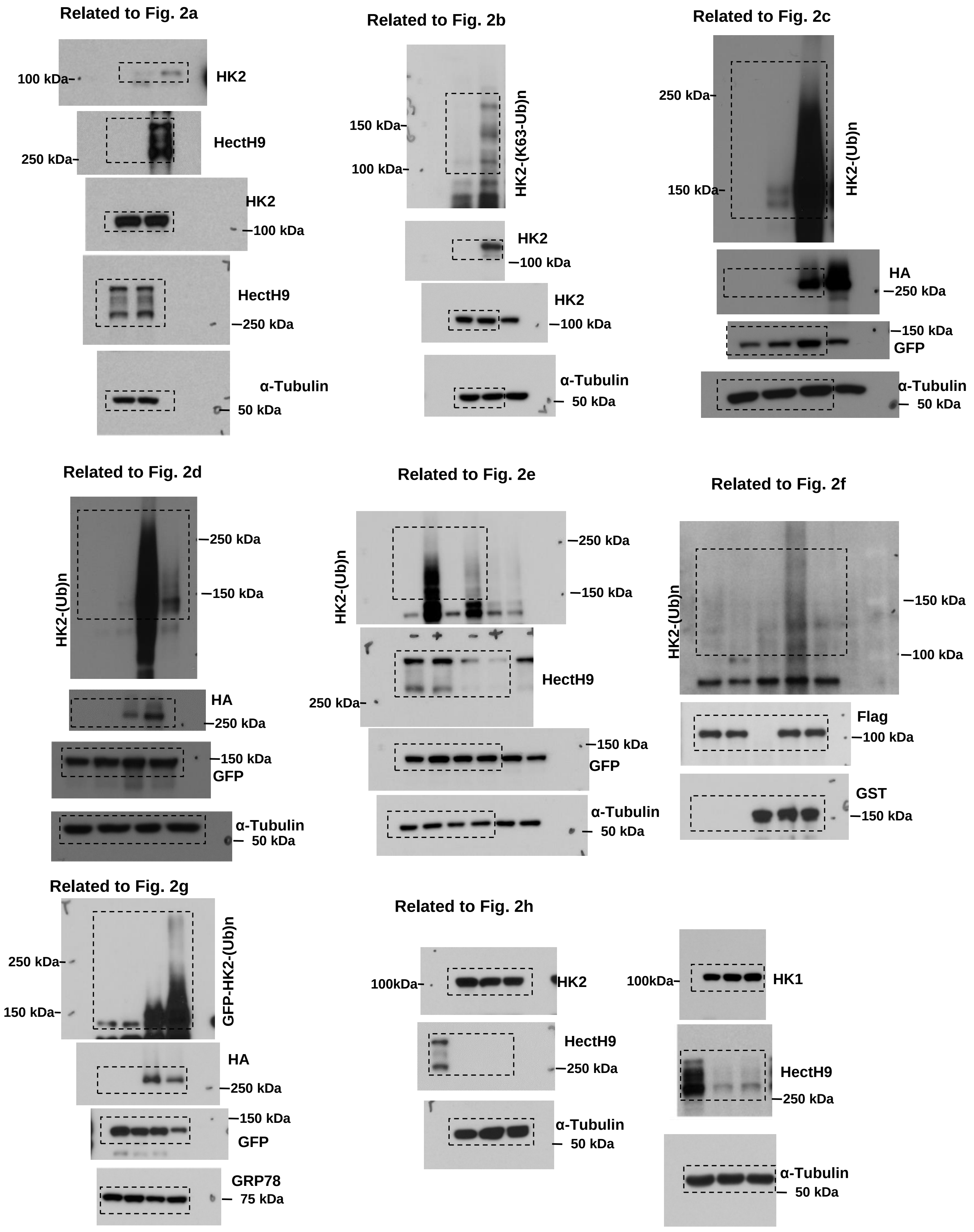

Related to Fig. 3b

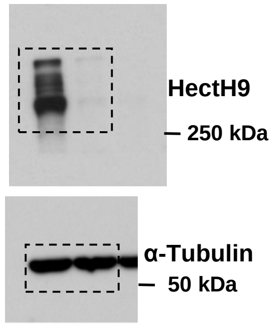

Related to Fig. 3c

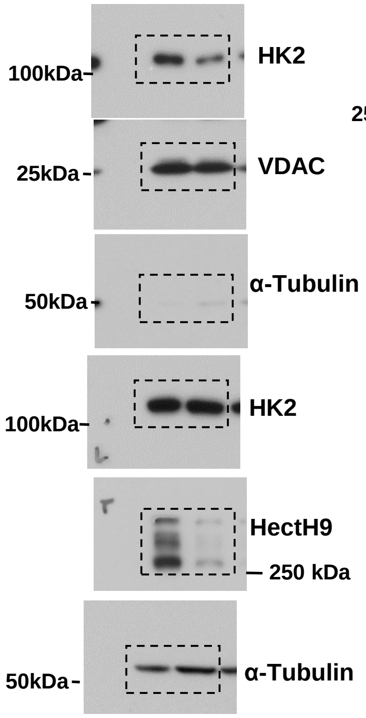

Related to Fig. 3e

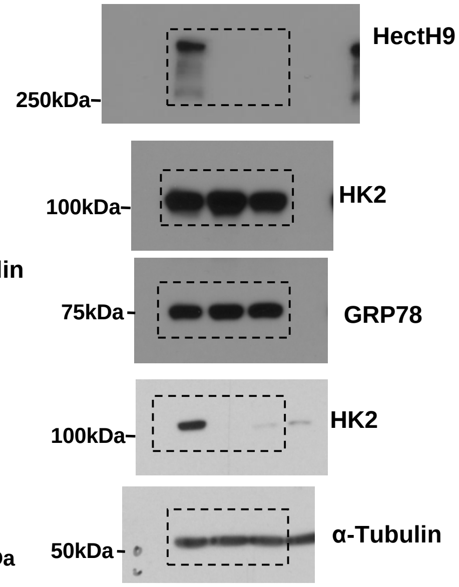

Related to Fig. 3h

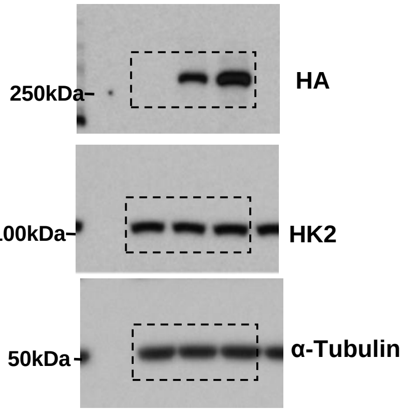

Related to Fig. 5a

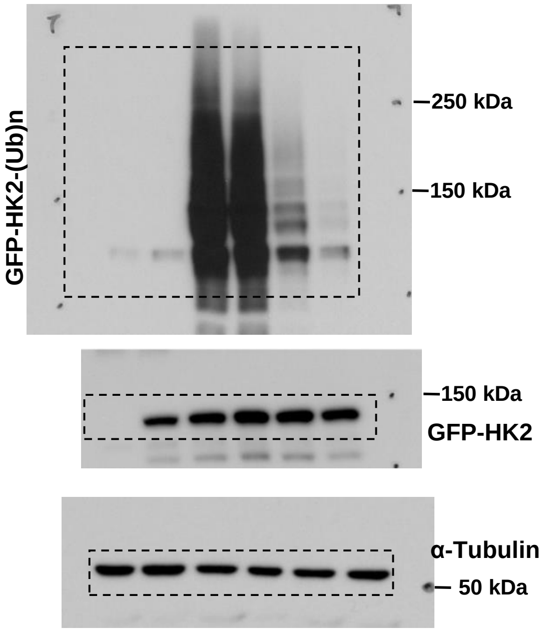

Related to Fig. 4a

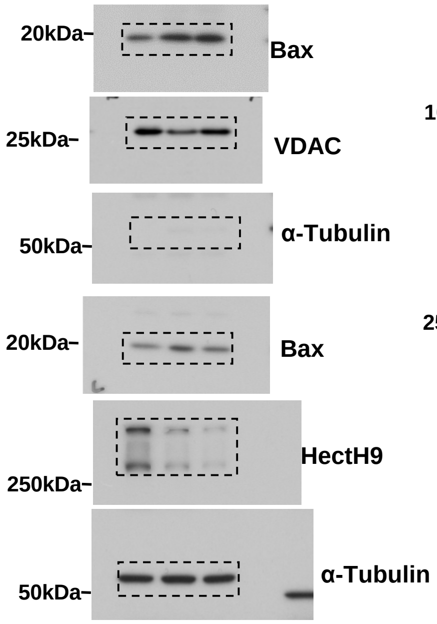

Related to Fig. 4b

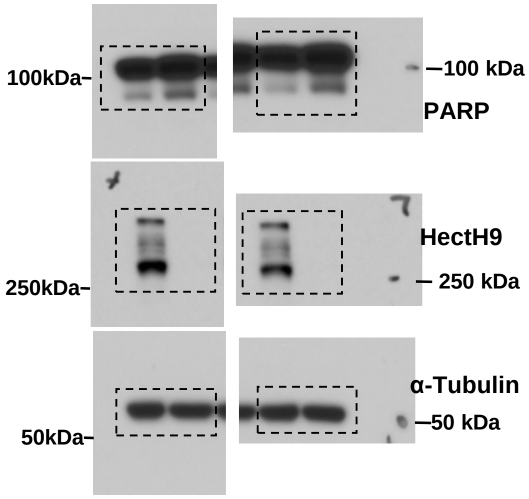

Related to Fig. 7g

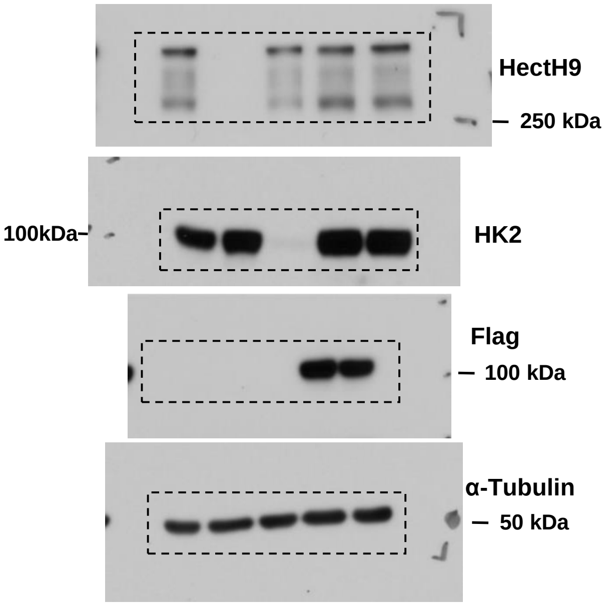

Related to Fig. 5c

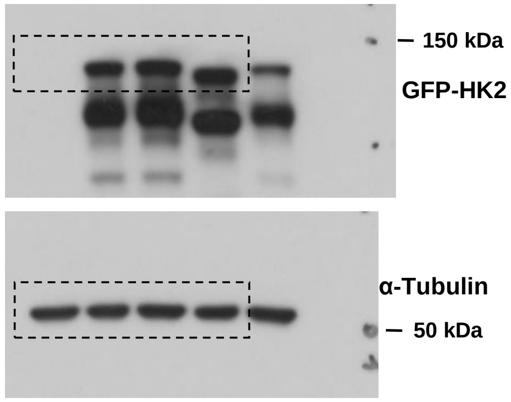

Supplementary Table 1. Antibodies used in this study

Primary antibody	Source	Cat. No.	Dilution
Hexokinase 1 (HK1)	Cell Signaling (Danvers, MA, USA)	#2024	1:5,000
Hexokinase 2 (HK2)-WB	Santa Cruz Biotechnology (Dallas, TX, USA)	sc-374091	1:5,000
Hexokinase 2 (HK2)-IF	Santa Cruz Biotechnology (Dallas, TX, USA)	sc-374091	1:100
Hexokinase 2 (HK2)-IP	Santa Cruz Biotechnology (Dallas, TX, USA)	sc-374091	2μg/mg lysate
Hexokinase 2 (HK2)-IHC	Cell Signaling (Danvers, MA, USA)	#2867	1:50
HectH9 (Arf BP1)	Abcam (Cambridge, MA, USA)	ab70161	1:2,000
HectH9 (Arf BP1)-IHC	Abcam (Cambridge, MA, USA)	ab70161	1:200
HectH9 (Lasu1)-IP	Bethyl Lab (Montgomery, TX, USA)	A300-486A	2μg/mg lysate
VDAC	Millipore (Burlington, MA, USA)	Ab10527	1:10,000
VDAC-IF	Millipore (Burlington, MA, USA)	Ab10527	1:300
PARP-1	Santa Cruz Biotechnology (Dallas, TX, USA)	sc-8007	1:4,000
Bax	Santa Cruz Biotechnology (Dallas, TX, USA)	sc-493	1:1,000
p53	Santa Cruz Biotechnology (Dallas, TX, USA)	sc-126	1:10,000
HIF-1a	BD Transduction Laboratories (San Jose, CA, USA)	#610958	1:1,000
Myc	SIGMA (Milwaukee, MI, USA)	11667203001	1:5,000
GST	Cell Signaling (Danvers, MA, USA)	#2622	1:5,000
Flag	SIGMA (Milwaukee, MI, USA)	F1804	1:2,000
HA	BioLegend (San Diego, CA, USA)	901514	1:5,000
GFP-WB	Cell Signaling (Danvers, MA, USA)	#2555	1:2,000
GFP-IP	ThermoFisher Scientific (Rockford, IL, USA)	A-11122	1μg Ab/mg lysate
GRP78	BD Transduction Laboratories (San Jose, CA, USA)	#610978	1:10,000
α-Tubulin	SIGMA (Milwaukee, MI, USA)	T6074	1:10,000
β-Actin	SIGMA (Milwaukee, MI, USA)	A1978	1:20,000
GAPDH	Santa Cruz (Dallas, TX, USA)	sc-48167	1: 5,000
K63-linkage polyubiquitin	Cell Signaling (Danvers, MA, USA)	#5621	1:1,500
K48-linkage polyubiquitin	Cell Signaling (Danvers, MA, USA)	#4289	1:1,000
Phospho-Akt Substrate (RXXS*/T*)	Cell Signaling (Danvers, MA, USA)	#9614	1:500
