# Supplementary material for: Non-proteolytic ubiquitination of Hexokinase 2 by HectH9 controls tumor metabolism and cancer stem cell expansion
Source: Nat Commun. 2019 Jun 14;10:2625. doi: 10.1038/s41467-019-10374-y (PMC6573064; doi:10.1038/s41467-019-10374-y)
Supplement: Supplementary file 2 — Reporting Summary [file 41467_2019_10374_MOESM2_ESM.pdf]

nature research

Corresponding author(s): NCOMMS-17-29861

Last updated by author(s): May 3, 2019

Reporting Summary

Nature Research wishes to improve the reproducibility of the work that we publish. This form provides structure for consistency and transparency in reporting. For further information on Nature Research policies, see Authors & Referees and the Editorial Policy Checklist.

Statistics

For all statistical analyses, confirm that the following items are present in the figure legend, table legend, main text, or Methods section.

n/a	Confirmed	
☐	☒	The exact sample size (*n*) for each experimental group/condition, given as a discrete number and unit of measurement
☐	☒	A statement on whether measurements were taken from distinct samples or whether the same sample was measured repeatedly
☐	☒	The statistical test(s) used AND whether they are one- or two-sided<br>*Only common tests should be described solely by name; describe more complex techniques in the Methods section.*
☒	☐	A description of all covariates tested
☒	☐	A description of any assumptions or corrections, such as tests of normality and adjustment for multiple comparisons
☐	☒	A full description of the statistical parameters including central tendency (e.g. means) or other basic estimates (e.g. regression coefficient) AND variation (e.g. standard deviation) or associated estimates of uncertainty (e.g. confidence intervals)
☒	☐	For null hypothesis testing, the test statistic (e.g. F, t, r) with confidence intervals, effect sizes, degrees of freedom and P value noted<br>*Give P values as exact values whenever suitable.*
☒	☐	For Bayesian analysis, information on the choice of priors and Markov chain Monte Carlo settings
☒	☐	For hierarchical and complex designs, identification of the appropriate level for tests and full reporting of outcomes
☐	☒	Estimates of effect sizes (e.g. Cohen's *d*, Pearson's *r*), indicating how they were calculated

Our web collection on statistics for biologists contains articles on many of the points above.

Software and code

Policy information about availability of computer code

Data collection	The TCGA data were retrieved from cBioPortal (http://www.cbioportal.org/).
Data analysis	Photoshop(Adobe), ImageJ (NIH), NIS-Element (Nikon) and Graphpad Prism sofrwares were used in the study.

For manuscripts utilizing custom algorithms or software that are central to the research but not yet described in published literature, software must be made available to editors/reviewers. We strongly encourage code deposition in a community repository (e.g. GitHub). See the Nature Research guidelines for submitting code & software for further information.

Data

Policy information about availability of data

All manuscripts must include a data availability statement. This statement should provide the following information, where applicable:

- Accession codes, unique identifiers, or web links for publicly available datasets
- A list of figures that have associated raw data
- A description of any restrictions on data availability

Data generated from this study are included in this article and its Supplementary Information files or will be provided from the corresponding author upon reasonable request. Uncropped scans for blots are presented in Supplementary Fig. 13.

Field-specific reporting

Please select the one below that is the best fit for your research. If you are not sure, read the appropriate sections before making your selection.

☒ Life sciences ☐ Behavioural & social sciences ☐ Ecological, evolutionary & environmental sciences

For a reference copy of the document with all sections, see nature.com/documents/nr-reporting-summary-flat.pdf

Life sciences study design

All studies must disclose on these points even when the disclosure is negative.

Sample size	The chosen sample size are based on the numbers used for previous publications, which is most optimal to generate statistically significant results. All statistical significance was determined by two-tailed Student's t tests, and p values less than 0.05 were considered statistically significant. The exact n values for each experiment can be found in the figure legends. For in vivo tumorigenesis experiments, the mouse number was determined by power analysis based on the fact that with a sample size of five and with a two-sided type I error rate of 0.05, the study would have 90% power to detect a 40% difference.
Data exclusions	No were data were excluded.
Replication	All replication attempts were successful. The number of times an experiment was repeated (biological replicates) and exact n values are indicated in the figure legends and methods section.
Randomization	The samples/cells were randomized to be examined. For in vivo tumorigenesis assays, mice were randomly grouped for tumor cell inoculation.
Blinding	Blinding was not relevant to the study because all cells/samples were analyzed in the same way. For in vivo tumorigenesis assays, blinding was not done during tumor inoculation but during the measurement of tumor size.

Reporting for specific materials, systems and methods

We require information from authors about some types of materials, experimental systems and methods used in many studies. Here, indicate whether each material, system or method listed is relevant to your study. If you are not sure if a list item applies to your research, read the appropriate section before selecting a response.

Materials & experimental systems

n/a	Involved in the study	
☐	☒	Antibodies
☐	☒	Eukaryotic cell lines
☒	☐	Palaeontology
☐	☒	Animals and other organisms
☒	☐	Human research participants
☒	☐	Clinical data

Methods

n/a	Involved in the study	
☒	☐	ChIP-seq
☐	☒	Flow cytometry
☒	☐	MRI-based neuroimaging

Antibodies

Antibodies used	All antibodies are commercial. The reference and dilution is described in Supplementary Table 1.
Validation	Antibody validations were performed as described on the manufacturers' websites and were supported by multiple publications.

Eukaryotic cell lines

Policy information about cell lines

Cell line source(s)	PC-3, MDA-MB-231, HeLa and 293T cells were obtained from ATCC.
Authentication	The cell lines were authenticated by short tandem repeat profiling by ATCC.
Mycoplasma contamination	The cell lines were verified to be free of mycoplasma contamination by the R&D MycoProbe® Mycoplasma Detection Kit.
Commonly misidentified lines (See ICLAC register)	No commonly misidentified lines were used in this study.

Animals and other organisms

Policy information about studies involving animals; ARRIVE guidelines recommended for reporting animal research

Laboratory animals	5- to 6-week-old athymic nude mice (NCr-Foxn1nu) were purchased from Envigo.
Wild animals	The study did not involve wild animals.

Field-collected samples	The study did not involve sample collected from the field.
Ethics oversight	All research involving animals was complied with protocols approved by the Animal Care and Use Committee at Stony Brook University.

Note that full information on the approval of the study protocol must also be provided in the manuscript.

Flow Cytometry

Plots

Confirm that:

☒ The axis labels state the marker and fluorochrome used (e.g. CD4-FITC).

☒ The axis scales are clearly visible. Include numbers along axes only for bottom left plot of group (a 'group' is an analysis of identical markers).

☒ All plots are contour plots with outliers or pseudocolor plots.

☒ A numerical value for number of cells or percentage (with statistics) is provided.

Methodology

Sample preparation	Sample preparation is described in the Methods.
Instrument	FACSCalibur, LSRFortessa, BD Biosciences
Software	BD CellQuest, BD FACSDIVA 8.0.1
Cell population abundance	Cells were analyzed without sorting
Gating strategy	For all flow cytometry experiments, on the SSC/FSC plots the dense population on both axes was used to select for single cells. For determining Annexin V+/PI- cells for cellular apoptosis, an unstained sample, a sample with Annexin V staining and a sample with PI staining were used to set up the gating strategy. For determining ALDH-positive cells, an unstained sample and samples added with the ALDH inhibitor DEAB were used to set up the gating strategy. For determining CellROX green-positive cells, an unstained sample was used as a negative control for baseline fluorescence. Gating strategy is presented in Figure 6a, Figure S3 and Figure S5.

☒ Tick this box to confirm that a figure exemplifying the gating strategy is provided in the Supplementary Information.
